# New Thalidomide-Resembling Dicarboximides Target ABC50 Protein and Show Antileukemic and Immunomodulatory Activities

**DOI:** 10.3390/biom9090446

**Published:** 2019-09-04

**Authors:** Marcin Cieślak, Julia Kaźmierczak-Barańska, Karolina Królewska-Golińska, Mariola Napiórkowska, Iga Stukan, Urszula Wojda, Barbara Nawrot

**Affiliations:** 1 Centre of Molecular and Macromolecular Studies, Polish Academy of Sciences, 112 Sienkiewicza Str., 90-363 Lodz, Poland; 2 Department of Medical Chemistry, Medical University of Warsaw, 3 Oczki Str., 02-007 Warsaw, Poland; 3 Laboratory of Preclinical Testing of Higher Standard, Nencki Institute of Experimental Biology of Polish Academy of Sciences, 02-093 Warsaw, Poland

**Keywords:** apoptosis, dicarboximides, anticancer compounds, cytotoxicity, leukemia, ABC50 (ABCf1), protac (proteolysis targeting chimera)

## Abstract

We identified novel dicarboximides that were selectively cytotoxic towards human leukemia cells. Using chemical and biological methods, we characterized the biological activity, identified cellular protein targets and defined the mechanism of action of the test dicarboximides. The reported IC_50_ values (concentration required to reduce cell survival fraction to 50% of control) of selected dicarboximides were similar or lower than IC_50_ of registered anticancer drugs, for example cytarabine, sorafenib, irinotecan. Test compounds induced apoptosis in chronic myelogenous (K562) and acute lymphoblastic (MOLT-4) leukemia cells by activation of receptor and mitochondrial apoptotic pathways and increased the expression of proapoptotic genes (*BAX, NOXA, HTRA2, TNFRSF10B, ESRRBL1)*. Selected dicarboximides displayed immunomodulatory activity and downregulated IKZF1 and IKZF3 transcription factors in K562 and MOLT-4 leukemia cells. ATP-binding cassette protein 50 (ABC50) was identified as a target for dicarboximides. Cancer cells with knocked down ABC50 showed increased resistance to dicarboximides. Based on the structure of dicarboximides and thalidomide, novel proteolysis-targeting chimeras (PROTACs) were synthesized and used as tools to downregulate ABC50 in leukemia cells.

## 1. Introduction

Cancer diseases with quickly rising mortality rates constitute nowadays the major problem of clinical medicine. There is an ongoing need for new anticancer drugs with improved pharmacological profile. While the design of new drugs would be facilitated by development of more detailed knowledge of genetic and molecular mechanisms of cancer pathogenesis, many of them are derivatives of already known therapeutics. Effective anticancer therapy should not only inhibit proliferation of cancer cells but also induce their apoptosis. Apoptosis, or programmed cell death, is a metabolic process used by an organism for removal of harmful, unnecessary or damaged cells in a controlled way. A proper balance between cell proliferation and apoptosis maintains the optimal numbers and types of cells during development as well as in mature tissues. Cancer cells can escape these maintenance processes, so there is a growing demand for methods to get them back on track of controlled apoptotic elimination. In principle, there are two pathways leading to apoptosis: (1) receptor mediated (extrinsic, utilizing extracellular signals sensed by death receptors), and (2) mitochondrial (intrinsic). Sometimes these pathways overlap leading to the amplification of proapoptotic signals [1,2,3,4]. Key enzymes involved in apoptosis are cysteine proteases called caspases, which operate either as initiators (caspases 8, 9, 10) or effectors (caspases 3, 6 and 7). The latter are responsible for cell disintegration during the final stage of apoptosis [5,6]. Necrosis, in contrast to apoptosis, is an unregulated premature cell death upon an injury, detrimental to cells.

Numerous reports show that dicarboximides exhibit the anti-neoplastic activity. The first clinical trials of naphthalimide derivatives (amonafide and mitonafide) as antineoplastic compounds were carried out in the 1980s [7,8]. Although in the phases II and III these compounds showed anticancer activity in leukemias and solid tumors, they were abandoned because of serious side effects. The cytotoxicity of these compounds resulted mainly from intercalation into DNA and inhibition of topoisomerase II activity [9,10]. Another interesting class of compounds consists of indolomaleimide derivatives, which exert anticancer activity by intercalation to DNA, induction of apoptosis, cell-cycle arrest and inhibition of protein kinase C [11,12]. Thus, dicarboximides have interesting biological properties and may affect a variety of biological processes, with undisputable potential as anticancer drugs.

Thalidomide and its derivatives lenalidomide and pomalidomide are probably the most recognized representatives of dicarboximides. They are collectively known as immunomodulatory drugs (IMiDs) and display a wide range of biological activities including inhibition of angiogenesis, induction of oxidative stress, cytotoxicity and immunomodulation. IMiDs are currently used for treatment of hematological malignancies, particularly multiple myeloma [13]. From chemical perspective these compounds are composed of phthalimide and glutarimide moieties. One of the possible mechanisms of action of IMiDs as anti-leukemia (particularly anti-myeloma) agents has been identified and is based on selective targeting of cellular proteins for ubiquitination by cullin-4 RING E3 ligase (CRL4). Ubiquitinated proteins are subsequently degraded by a proteasome. It was demonstrated that IMiDs bind cereblon (CRBN), a protein that is a part of the CRL4 complex [14,15]. Binding of IMiDs to CRBN results in enhanced ubiquitination and degradation of two zinc finger transcription factors: IKZF1 (Ikaros family zinc finger protein 1, Ikaros) and IKZF3 (Ikaros family zinc finger protein 3, Aiolos). These transcription factors are necessary for myeloma cells survival thus their degradation is lethal for myeloma cells. Interestingly, IMiDs-driven downregulation of IKZF1/IKZF3 is rather non-toxic to T-cells or T-cell leukemias but results in increased expression of interleukin 2 (IL-2) and decreased expression of tumor necrosis factor alpha (TNF-α) in monocytes [16,17].

IKZF1 and IKZF3 are indispensable for development of lymphoid tissue, particularly T- and B-lymphocytes and natural killer (NK) cells. IKZF1 is expressed early during the formation of lymphoid system while IKZF3 is expressed in the middle and late stages of T- and B-cell maturation. Interestingly, Ikaros is necessary for pluripotent stem cells differentiation not only to lymphoid but also myeloid/erythroid lineages. Studies in mice showed that deletion of DNA binding domain of Ikaros resulted in generation of abnormal T-cells and was lethal to T-cell leukemias [18]. These data suggest that downregulation of Ikaros and/or Aiolos either by low molecular weight organic compounds or by genetic modifications can have cytotoxic effects on hematological malignancies including T-cell and myeloid/erythroid leukemias.

In the present study we investigated pro-apoptotic properties and the mechanism of cytotoxicity of new derivatives of dicarboximides. These compounds were earlier found to be remarkably cytotoxic and selective towards human leukemia cells (K562, HL-60), and non-toxic to adherent cancer cells (HeLa, human cervix carcinoma) and to primary endothelial cells (HUVEC, human umbilical vein endothelial cells) [19,20]. We identified cellular proteins targeted by these compounds and investigated their immunomodulatory activity. 

The complete list of dicarboximides, biological tests and cell lines is included in the supplementary material (Table S1).

## 2. Materials and Methods 

### 2.1. Chemistry

All solvents, reagents and chemicals used in these studies were purchased from Aldrich Chemical and Merck AG. Melting points were determined with Electrothermal 9100 capillary apparatus and were uncorrected. The nuclear magnetic resonance spectra were recorded in deuterated D6 dimethyl sulfoxide (DMSO-d_6_) or CDCl_3_ on VMNRS300 operating at 300 MHz (^1^H nuclear magnetic resonance (NMR)) and 75 MHz (^13^C-NMR). Chemical shifts (δ) are expressed in parts per million relatives to tetramethylsilane used as the internal reference. Coupling constants (J) values are given in hertz (Hz) and spin multiples are given as s (singlet), d (dublet), t (triplet), m (multiplet). Mass spectral ESI (Electrospray Ionization) measurements were carried out on a MicrOTOF II (Bruker, Bremen, Germany) instrument with TOF detector. The spectra were obtained in the positive ion mode. Flash chromatography was performed on Kieselgel (Merck, Darmstadt, Germany) 0.05–0.2 mm reinst (70–325 mesh ASTM) silica gel using chloroform as eluent. Progress of the reactions described in experimental section was monitored by TLC (Thin Layer Chromatography) on silica gel (plates with fluorescent indicator 254 nm, layer thickness 0.2 mm, Kieselgel G (Merck, Darmstadt, Germany), using chloroform-methanol as an eluent system at the *v/v* ratio of 9:1 or 9.5:0.5.

Dicarboximides **1**–**6** (Table 1) were synthesized according to previously reported procedures [20,21]. Compounds **6a**, **6b**, **7**–**9** were synthesized as described below. 

#### 2.1.1. Synthesis of 1,7,8,9-tetraphenyl-4-azatricyclo[5.2.1.0^2,6^]dec-8-ene-3,5-dione (**D**)

The starting imide **D** was prepared by the methods described earlier [20]. A mixture of 1,2,3,4-tetraphenylcyclopenta-1,3-diene (0.02 mol) and maleimide (0.022 mol) was heated for 6 h in 20 mL of benzene. When the reaction was completed the solvent was evaporated under reduced pressure and the solid residue was crystallized from benzene.

Molecular weight (M.W.) = 467.5571; C_33_H_25_NO_2_; Yield: 81%, white powder, m. p. 246.5–246.9 °C (from benzene); ^1^H NMR (300 MHz, DMSO-*d_6_*^+^ TMS (tetramethylsilane), δ/ppm): 11.44 (1H, *s*, NH), 7.74 (4H, *d*, *J* = 7.2, H-aromatic), 7.24 (6H, m, H-aromatic), 6.88 (6H, m, H-aromatic), 6.60 (4H, m, H-aromatic), 4.20 (2H, s, C2-H, C6-H), 3.23 (1H, *d*, *J* = 8.7, C10-H), 2.23 (1H, *d*, *J* = 8.4, C10-H); ^13^C-NMR (DMSO, 75 MHz) δ (ppm): 178.0 (2C-3, 5), 145.0 (2C-11, 11′), 139.8 (2C-15, 15′), 134.5 (2C-8, 9), 129.6 (4C-13, 13′), 129.1 (4C-16, 16′), 127.7 (4C-12, 12′), 127.1 (4C-17, 17′), 126.6 (2C-18, 18′), 126.4 (2C-14, 14′), 65.1 (C-10), 62.9 (2C-1, 7), 52.9 (2C-2, 6). High Resolution Mass Spectrometry (HRMS, *m/z*): calculated value for [M + Na]^+^ 100% = 490.1778; found 100% = 490.1777, 35.6% = 491.1817, 6.6% = 492.1856.

#### 2.1.2. Synthesis of 4-[-3-(amino)-2-hydroxypropyl]-1,7,8,9-tetraphenyl-4-azatricyclo[5.2.1.0^2,6^]dec-8-ene-3,5-dione (**6a**)

**Scheme 1:**
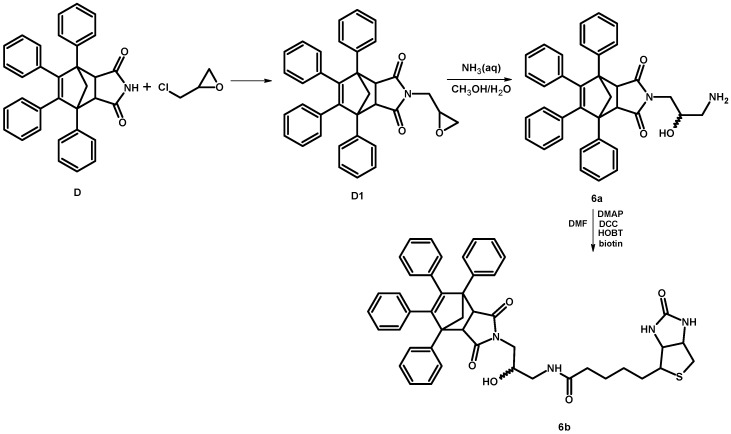
Synthesis of derivative **6a** and biotinylated derivative **6b**.

The amino alkanol derivative **6a** was obtained in two-step reaction (Scheme 1) according to the method described previously [20].

Step I: the mixture of 1,7,8,9-tetraphenyl-4-azatricyclo[5.2.1.0^2,6^]dec-8-ene-3,5-dione (**D**) (0.01 mol) with 1-chloro-2,3-epoxypropane (50 mL), in the presence of an anhydrous K_2_CO_3_ (0.01 mol) was stirred on a magnetic stirrer under reflux with a tube with CaCl_2_. The reaction was carried out at room temperature, for 15 h. When the reaction was completed (TLC control) the inorganic precipitate was filtered off and the filtrate was concentrated. The obtained oily product was purified by column chromatography on silica gel (eluent: chloroform and chloroform: methanol; 50:0.2, *v/v*) to deliver pure derivative **D1**.

Step II: to the solution of 4-(oxirane-2-ylmethyl)-1,7,8,9-tetraphenyl-4 azatricyclo[5.2.1.0^2,6^]dec-8-ene-3,5-dione (**D1**) (0.001 mol) in a mixture of methanol/water (10:1 *v/v*) ammonium hydroxide (concentrated, 5 mL) was added. Reactions were carried out at room temperature on a magnetic stirrer under reflux for 72 hours. When the reaction was completed (TLC control), the excess of the solvent was evaporated, and the crude product was purified by column chromatography on silica gel (eluent: chloroform or chloroform: methanol; 50:0.2, 50:0.5, *v/v*). 

*4-(oxiran-2-ylmethyl)-1,7,8,9-tetraphenyl-4-azatricyclo[5.2.1.0^2,6^]dec-8-ene-3,5-dione* (**D1**)

M.W. = 523.6203; C_36_H_29_NO_3;_ Yield: 52%; white powder, m.p. 229–230 °C (from MeOH); ^1^H-NMR (300MHz, CDCl_3_, δ/ppm): 7.70 (4H, m, H-aromatic), 7.28 (6H, m, H-aromatic), 6.93 (6H, m, H-aromatic), 6.55 (4H, m, H-aromatic), 4.18 (2H, *s*, C2-H, C6-H), 3.78 (1H, m, C19-H), 3.46 (1H, m, C19-H), 3.16 (1H, d, *J* = 9.0, C10-H), 3.07 (1H, m, C20-H), 2.64 (2H, m, C21-H), 2.34 (1H, d, *J* = 9.0, C10-H); ^13^C NMR (CDCl_3_, 75 MHz) δ (ppm): 176.4 (2C-3, 5), 145.1 (2C-11, 11′), 139.7 (2C-15, 15′), 134.2 (2C-8, 9), 129.4 (4C-13, 13′), 129.1 (4C-16, 16′), 127.8 (4C-12, 12′), 127.2 (4C-17, 17′), 126.7 (2C-18, 18′), 126.5 (2C-14, 14′), 65.2 (C-10), 63.0 (2C-1, 7), 52.1 (2C-2, 6), 51.8 (C-20), 48.2 (C-19), 45.3 (C-21); HRMS (*m/z*): calculated value for [M + Na]^+^ 100% = 546.2410; found 100% = 546.2901, 10% = 547.3011.

*4-[-3-(amino)-2-hydroxypropyl]-1,7,8,9-tetraphenyl-4-azatricyclo[5.2.1.0^2,6^]dec-8-ene-3,5-dione* (**6a**)

M.W. = 540.6508 (free amine); C_36_H_32_N_2_O_3;_ Yield: 34%; oil; ^1^H-NMR (300 MHz, CDCl_3_, δ/ppm): 7.70 (4H, m, H-aromatic), 7.25 (5H, m, H-aromatic), 6.89 (7H, m, H-aromatic), 6.56 (4H, m, H-aromatic), 4.17 (2H, m, C2-H, C6-H), 3.80 (1H, m, C20-H), 3.57 (2H, m, C19-H), 3.16 (1H, m, C10-H), 2.78 (1H, m, C21-H), 2.62 (1H, m, C21-H), 2.34 (1H, m, C10-H); ^13^C NMR (CDCl_3_, 75 MHz) δ (ppm): 177.1 (C-3/*R/S*-20), 177.0 (C-5/*R/S*-20), 145.2 (C-11/*S*-20), 145.1 (C-11/*R*-20), 144.9 (C-11′/*S*-20), 144.9 (C-11′/*R*-20), 139.3 (C-15/*S*-20), 139.3 (C-15/*R*-20), 139.2 (C-15′/*S*-20), 139.2 (C-15′/*R*-20), 134.0 (C-8/*S/R*-20), 133.9 (C-9/*S*-20), 133.8 (C-9/*R*-20), 129.9 (2C-13/*S*-20), 129.9 (2C-13/*R*-20), 129.8 (2C-13′/*S*-20), 129.8 (2C-13′/*R*-20), 129.1 (4C-16′, 16), 128.0 (4C-12′, 12), 127.4 (2C-17′), 127.3 (2C-17), 127.1 (C-18), 127.0 (C-18′), 126.8 (2C-14, 14′), 69.4 (C-20), 65.9 (C-10/*R*-20), 65.8 (C-10/*S*-20), 63.6 (C-7/*S*-20), 63.6 (C-7/*R*-20), 63.6 (C-1/*S*-20), 63.5 (C-1/*R*-20), 52.2 (C-6/*S*-20), 52.2 (C-6/*R*-20), 49.5 (C-2), 44.4 (C-21), 42.8 (C-19); HRMS (*m/z*): calculated value for [M + H]^+^ 100% = 541.2486; found 100% = 541.2486, 53.4% = 542.2501, 10% = 543.2530.

#### 2.1.3. Synthesis of Biotinylated Derivative **6b**

To a mixture of 0.795 mmol (430 mg) of derivative **6a**, dicyclohexylcarbodiimide (DCC, 164 mg, 0.795 mmol, N,N-dimethylaminopyridine (DMAP, 9.71 mg, 0.0795 mmol) and hydroxybenzotriazole (HOBT, 10.7 mg, 0.0795 mmol), dissolved in N,N-dimethylformamide (DMF, 20 mL), a solution of 0.795 mmol of biotin (194 mg) in DMF (12 mL) was slowly added dropwise and the reaction mixture was stirred for 24 h at room temperature. Then the solvent was removed under reduced pressure. The reaction mixture was poured at the silica gel column and the desired product was eluted with the gradient of methanol (0 to 10%) in chloroform. The numbering of atoms of derivative **6b** used in the description of nuclear magnetic spectra is available in supplementary material (Figure S1).

*N-[2-hydroxy-3-(1,7,8,9-tetraphenyl)-3,5-dioxo-4-azatricyclo[5.2.1.0^2,6^]dec-8-en-4-yl)propyl]-5-(2-oxo hexahydro-1H-thieno[3,4-d]imidazol-4-yl)pentanamide* (**6b**)

M.W. = 766.3188; C_46_H_46_N_4_O_5_S_;_ Yield: 54,5%; oil; ^1^H-NMR (300 MHz, CDCl_3_, δ/ppm): 7.66 (4H, m, H-aromatic), 7.26 (4H, m, H-aromatic), 7.14 (2H, m, H-aromatic), 6.94 (2H, m, H-aromatic), 6.84 (4H, m, H-aromatic), 6.52 (4H, m, H-aromatic), 6.31 (1H, m, NH-biot), 5.01 (1H, m, NH-biot), 4.34 (1H, m, C28-H), 4.16 (3H, m, C29-H, C19-H), 4.00 (1H, m, C20-H), 3.52 (3H, m, C27-H, C30-H), 3.32 (1H, m, C2-H), 3.06 (2H, m, C21-H), 2.73 (1H, m, C10-H), 2.54 (1H, m, C6-H), 2.30 (1H, m, C10-H), 2.17 (2H, m, C23-H), 1.61 (4H, m, C24-H, C26-H), 1.39 (2H, m, C25-H). ^13^C NMR (CDCl_3_, 75 MHz) δ (ppm): 177.2 (C-3/*R/S*-20), 177.2 (C-5/*R*-20), 177.1 (C-5/*S*-20), 174.3 (C-22/*S*-20), 174.2 (C-22/*R*-20), 164.8 (C-31/*S*-20), 164.7 (C-31/*R*-20), 145.3 (C-11/*S*-20), 145.2 (C-11/*R*-20), 144.9 (C-11′/*S*-20), 144.7 (C-11′/*R*-20), 139.3 (C-15/*S*-20), 139.3 (C-15/*R*-20), 139.2 (C-15′/*S*-20), 139.2 (C-15′/*R*-20), 134.1 (C-8/*S*-20), 134.0 (C-8/*R*-20), 133.9 (C-9/*S*-20), 133.8 (C-9/*R*-20), 129.9 (2C-13/*S*-20), 129.9 (2C-13/*R*-20), 129.8 (2C-13′/*S*-20), 129.8 (2C-13′/*R*-20), 129.1 (2C-16), 129.0 (2C-16′), 128.0 (4C-12′, 12), 127.3 (4C-17′, 17), 127.1 (C-18), 127.0 (C-18′), 126.7 (C-14), 126.7 (C-14′), 77.2 (C-20), 68.1 (C-10/*S*-20), 67.7 (C-10/*R*-20), 63.5 (C-7/*S*-20), 63.4 (C-7/*R*-20), 63.4 (C-1/*S*-20), 63.3 (C-1/*R*-20), 61.5 (C-28/*S*-20), 61.4 (C-28/*R*-20), 60.3 (C-29), 55.7 (C-27/*S*-20), 55.6 (C-27/*R*-20), 52.3 (C-6/*S*-20), 52.2 (C-6/*R*-20), 51.9 (C-2/*S*-20), 51.8 (C-2/*R*-20), 43.0 (C-21), 42.8 (C-19), 40.3 (C-30), 36.0 (C-23/*S*-20), 35.8 (C-23/*R*-20), 28.2 (C-25/*S*-20), 28.0 (C-25/*R*-20), 27.9 (C-26/*S*-20), 27.7 (C-26/*R*-20), 25.9 (C-24/*S*-20), 25.5 (C-24/*R*-20); HRMS (*m/z*): calculated value for [M + H]^+^ 100% = 767.3261; found 100% = 767.3260;

#### 2.1.4. Synthesis of Derivatives **7**–**9**

**Scheme 2:**
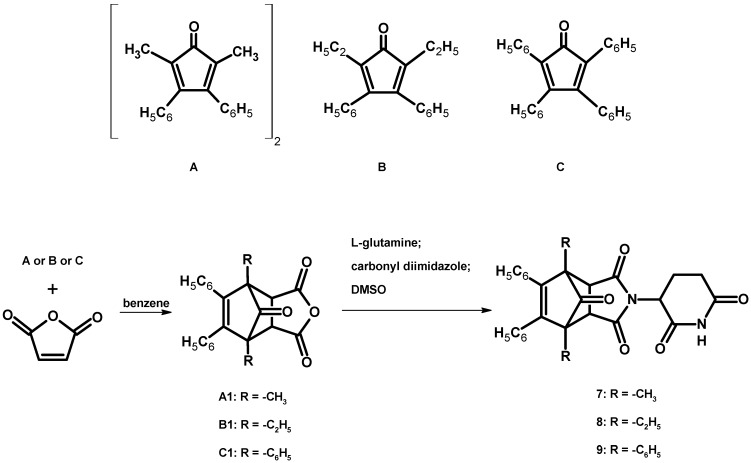
Synthesis of derivatives **7**–**9**.

The general synthesis of derivatives **7**–**9** is shown on Scheme 2. 

##### Synthesis of Anhydrides **A1**, **B1** and **C1**

An appropriate cyclopentadienone:

2,5-dimethyl-3,4-diphenylcyclopentadienone as a dimer (0.002 mol of dimer)
2,5-diethyl-3,4-diphenylcyclopenta-2,4-dienone (0.007 mol)
1,2,3,4-tetraphenylcyclopenta-1,3-dienone (0.007 mol)

was dissolved in benzene (50 mL). Next, maleic anhydride (0.008 mol) was added to the solution. The reaction mixture was refluxed for 48 h. When the reaction was complete (TLC control) the mixture was left at the room temperature for 12 h, obtaining solid product. The obtained crude product was crystallized from benzene to give the white solid. Finally, product was filtered and dried. 

*1,7-dimethyl-8,9-diphenyl-4-oxatricyclo[5.2.1.0^2,6^]dec-8-ene-3,5,10-trione* (**A1**)

M.W. = 358.1205; C23H18O4; Yield: 60%; white powder, m.p. 188–190 °C; ^1^H-NMR (300 MHz, CDCl3, δ/ppm): 11.10 (1H, s, -NH), 7.19 (6H, m, H-aromatic), 6.97 (4H, m, H-aromatic), 3.49 (2H, s, C2-H, C6-H), 1.58(6H, s, -CH_3_); ^13^C NMR (CDCl_3_, 75 MHz) δ (ppm): 197.5 (C-10), 169.6 (2C-3, 5), 142.5 (2C-8, 9), 132.0 (2C-12, 12′), 129.5 (4C-14, 14′), 128.2 (2C-15, 15′), 128.1 (4C-13, 13′), 56.5 (2C-1, 7), 49.0 (2C-2, 6), 11.8 (2C-11, 11′); HRMS (*m/z*): calculated value for [M + Na]^+^ 100% = 381.1097; found 100% = 381.1097, 25.3% = 382.1134.

*1,7-diethyl-8,9-diphenyl-4-oxatricyclo[5.2.1.0^2,6^]dec-8-ene-3,5,10-trione* (**B1**)

M.W. = 386.4397; C_25_H_22_O_4_; Yield: 75%; white powder, m.p. 146–147 °C; ^1^H NMR (300 MHz, DMSO-d_6_+ TMS, δ/ppm): 7.20 (6H, m, H-aromatic), 6.97 (4H, m, H-aromatic), 4.02 (2H, s, C2-H, C6-H), 2.09 ( 2H, m, -CH_2_-), 1.88 ( 2H, m, -CH_2_-), 0.87 (6H, *t*, *J* = 7.5, -CH_3_); ^13^C NMR (DMSO, 75 MHz) δ (ppm): 197.0 (C-10), 171.3 (2C-3, 5), 142.4 (2C-8, 9), 133.0 (2C-13, 13′), 129.0 (4C-15, 15′), 128.1 (2C-16, 16′), 127.7 (4C-14, 14′), 59.3 (2C-1, 7), 45.5 (2C-2, 6), 18.5 (2C-11, 11′) 8.8 (2C-12, 12′); HRMS (*m/z*): calculated value for [M + Na]^+^ 100% = 409.1410; found 100% = 409.1410, 28.3% = 410.1437.

*1,7,8,9-tetraphenyl-4-oxatricyclo[5.2.1.0^2,6^]dec-8-ene-3,5,10-trione* (**C1**)

M.W. = 482.5253; C_33_H_22_O_4_; Yield: 65%; white powder, m.p. 216–218 °C; ^1^H-NMR (300 MHz, CDCl_3_, δ/ppm): 7.64 (2H, m, H-aromatic), 7.40 (4H, m, H-aromatic), 7.24 (2H, m, H-aromatic), 6.93 (2H, m, H-aromatic), 6.91 (4H, m, H-aromatic), 6.74 (4H, m, H-aromatic), 4.46 (2H, s, C2-H, C6-H); ^13^C NMR (CDCl_3_, 75 MHz) δ (ppm): 194.1 (C-10), 169.1 (2C-3, 5), 142.3 (2C-8, 9), 132.0 (4C-11, 11′, 15, 15′), 131.5 (4C-16, 16′), 130.1 (4C-17, 17′), 130.0 (4C-13, 13′), 128.4 (2C-18, 18′), 128.2 (4C-12, 12′), 127.9 (2C-14, 14′), 65.0 (2C-1, 7), 46.6 (2C-2, 6); HRMS (*m/z*): calculated value for [M + Na]^+^ 100% = 505.1410; found 100% = 505.1410, 35.5% = 506.1431.

##### Synthesis of Derivatives **7**–**9**

l-glutamine (0.0056 mol) was suspended in DMSO (10 mL) at room temperature. Next, an appropriate anhydride (A1, B1, C1) was added and the solution was stirred at temperature 80 °C. When reaction was finished (TLC monitoring, developing system: chloroform: methanol 9:1 *v/v*) the mixture was cooled to 20 °C, filtered and poured into a round-bottom flask containing carbonyl-diimidazole (0.006 mol) in DMSO (6 mL) at 20 °C [22]. The resulting solution was heated to 85–90 °C and stirred at this temperature to the end of the reaction (TLC monitoring, developing system: chloroform: methanol 9:1 *v/v*). Then the solution was poured into cold water (about 5 °C) and the mixture was stirred at room temperature for 24 h. The precipitated solid was filtered, crystalized from methanol: water (1:4 *v/v*) system and dried overnight to give crystalline white product. In the biological experiments the derivatives **7**–**9** were used as mixtures of diastereoisomers. The numbering of atoms of compounds **7**–**9** used in the description of nuclear magnetic spectra is available in supplementary material (Figure S2).

*4-(2,6-dioxopiperidin-3-yl)-1,7-dimethyl-8,9-diphenyl-4-azatricyclo[5.2.1.0^2,6^]dec-8-ene-3,5,10-trione* (**7**)

M.W. = 468.3467; C_28_H_24_N_2_O_5_; Yield: 30%; white powder, m.p. 217–218 °C; ^1^H-NMR (300 MHz, DMSO-d_6_+ TMS, δ/ppm): 11.10 (1H, s, -NH), 7.19 (6H, m, H-aromatic), 7.01 (2H, m, H-aromatic), 6.91 (2H, m, H-aromatic), 5.01 (1H, m, C3′-H), 3.64 (2H, m, C2-H, C6-H), 2.85 (1 H, m, C5′-H), 2.56 (1 H, m, C5′-H), 2.38 (1H, m, C4′-H), 1.75 (1H, m, C4′-H), 1.43 (3H, s, -CH_3_), 1.38 (3H, s, -CH_3_); ^13^C NMR (DMSO, 75 MHz) δ (ppm): 198.5 (C-10), 175.1 (2C-3, 5), 172.4 (C-6′), 168.6 (C-2′), 141.4 (C-8), 140.9 (C-9), 133.0 (2C-12, 12′), 129.4 (4C-14, 14′), 127.9 (2C-15, 15′), 127.4 (4C-13, 13′), 79.17 (C-3′), 55.7 (2C-1, 7), 49.5 (2C-2, 6), 30.6 (C-5′), 21.4 (C-4′), 11.9 (2C-11, 11′); HRMS (*m/z*): calculated value for [M + Na]^+^ 100% = 491.1577; found 100% = 491.1576, 35% = 492.1584.

*4-(2,6-dioxopiperidin-3-yl)-1,7-diethyl-8,9-diphenyl-4-azatricyclo[5.2.1.0^2,6^]dec-8-ene-3,5,10-trione* (**8**)

M.W. = 496.1997; C_30_H_28_N_2_O_5_; Yield: 40%; white powder, m.p. 232–234 °C; ^1^H-NMR (300 MHz, DMSO-d_6_+ TMS, δ/ppm): 11.12 (1H, s, -NH), 7.17 (6H, m, H-aromatic), 7.00 (2H, m, H-aromatic), 6.92 (2H, m, H-aromatic), 5.04 (1H, m, C3′-H), 3.78 (2H, m, C2-H, C6-H), 2.84 (1 H, m, C5′-H), 2.80 (1 H, m, C5′-H), 2.50 (1H, m, C4′-H), 2.05 (2H, m, -CH_2_-), 1.87 (2H, m, -CH_2_-), 1.71 ( 1H, m, C4′-H), 0.88 (6H, *t*, *J* = 6.9 Hz, -CH_3_); ^13^C NMR (DMSO, 75 MHz) δ (ppm): 198.1(C-10), 175.5 (2C-3, 5), 172.4 (C-6′), 168.6 (C-2′), 141.8 (2C-8, 9), 133.4 (2C-13, 13′), 129.2 (4C-15, 15′), 127.1 (2C-16, 16′), 127.4 (4C-14, 14′), 59.3 (2C-1, 7), 49.6 (C-3′), 43.5 (2C-2, 6), 30.6 (C-5′), 21.4 (C-4′), 19.0 (2C-11, 11′), 9.0 (2C-12, 12′); HRMS (*m/z*): calculated value for [M + Na]^+^ 100% = 519.1890; found 100% = 519.1891, 30% = 520.1899.

*4-(2,6-dioxopiperidin-3-yl)-1,7,8,9-tetraphenyl-4-azatricyclo[5.2.1.0^2,6^]dec-8-ene-3,5,10-trione* (**9**)

M.W. = 592.1998; C_38_H_28_N_2_O_5_; Yield: 30%; white powder, m.p. 230–232 °C; ^1^H-NMR (300 MHz, DMSO-d_6_+ TMS, δ/ppm): 11.16 (1H, s, -NH), 7.69 (4H, m, H-aromatic), 7.36 (6H, m, H-aromatic), 6.91 (6H, m, H-aromatic), 6.78 (4H, m, H-aromatic), 5.16 (1H, m, C3′-H), 4.60 (2H, m, C2-H, C6-H), 2.85 (1 H, m, C5′-H), 2.81 (1 H, m, C5′-H), 2.50 (1H, m, C4′-H), 1.74 (1H, m, C4′-H); ^13^C NMR (DMSO, 75 MHz) δ (ppm): 194.8 (C-10), 174.6 (2C-3, 5), 172.4 (C-6′), 168.5 (C-2′), 141.6 (2C-8, 9), 132.9 (4C-11, 11′,15,15′), 132.7 (4C-16, 16′), 130.5 (4C-17, 17′), 129.8 (4C-13, 13′), 127.7 (2C-18, 18′), 127.4 (4C-12, 12′), 127.3 (2C-14, 14′), 79.16 (C-3′), 65.2 (2C-1, 7), 50.0 (2C-2, 6) 30.5 (C-5′), 21.3 (C-4′); HRMS (*m/z*): calculated value for [M + Na]^+^ 100% = 615.1890; found 100% = 615.1890, 30% = 616.1909, 6.25% = 617.1952.

### 2.2. Cell Culturing and 3-(4,5-dimethylthiazol-2-yl)-2,5-diphenyltetrazolium bromide (MTT) Cytotoxicity Assay

Human umbilical vein endothelial cells (purchased from Life Technologies, Carlsbad, CA, USA) were cultured according to the manufacturer’s instructions in Medium 200 supplemented with Low Serum Growth Supplement. 1 × 10^4^ cells were seeded on each well on a 96-well plate (Nunc, Roskilde, Denmark). The HeLa (human cervix carcinoma, cat. #93021013), CFPAC (human pancreatic adenocarcinoma, cat. #91112501), K562 (chronic myelogenous leukemia, cat. #89121407), HL-60 (acute myelogenous leukemia, cat. #98070106), and MOLT-4 (Human acute T lymphoblastic leukemia, cat. #85011413) cells were purchased from European Collection of Authenticated Cell Cultures (ECACC). They were cultured in an RPMI 1640 medium supplemented with antibiotics (streptomycin, penicillin) and 10% fetal calf serum (20% for HL60), in a 5% CO_2_-95% air atmosphere. 7 × 10^3^ cells were seeded on each well on a 96-well plate (Nunc). 24 hours later the cells were exposed to the test compounds. The aliquots of stock solutions of the test compounds (freshly prepared in DMSO) were added to the cell cultures to yield final concentrations of 1, 10^−1^, 10^−2^, 10^−3^, 10^−4^, and 10^−5^ mM. The concentration of DMSO in the cell culture medium was 1%. The IC_50_ value (the concentration of a test compound required to reduce the cell survival fraction to 50% of the control) was calculated from a dose-response curve and used as a measure of cellular sensitivity to a given treatment. The IC_50_ values are given as means (± standard deviation (SD)) from 2 independent experiments with 4 technical replicates. The cytotoxicity of all compounds was determined by the MTT (3-(4,5-dimethylthiazol-2-yl)-2,5-diphenyltetrazolium bromide) assay (Sigma, St. Louis, MO, USA). Briefly, after 24 h or 48 h of incubation with a given compound, the cells were treated with the MTT reagent and incubation was continued for 2 h. MTT-formazan crystals were dissolved in 20% SDS and 50% DMF at pH 4.7 and VIS light absorption was measured at 570 and 650 nm on a microplate reader FLUOstar Omega (BMG Labtech, Offenburg, Germany). As a control (100% viability), cells grown in the presence of 1% DMSO were used.

### 2.3. Activation of Caspase-3/7 and Caspase-8/9 Determined by Fluorescent and Luminescent Assays

K562 and MOLT-4 cells were cultured in a RPMI 1640 medium supplemented with antibiotics and 10% fetal bovine serum in a 5% CO_2_ atmosphere at 37 °C. 20 × 10^3^ cells were seeded on each well on a 96-well plate. After 24 h the cells were exposed to the test compounds at concentration of 5× IC_50_ for another 18 h (caspase 3 and 7), or 3 h and 6 h (caspase 8 and 9). The cells were also exposed to 1% DMSO (control) or 1μM staurosporine (a strong inducer of cell apoptosis, Sigma, St. Louis, MO). The induction of cell apoptosis was analyzed by Apo-ONE^®^ Homogeneous Caspase-3/7 Assay (Promega, Madison, WI, USA). After 18 h of incubation with the test compounds, the cells were treated with the caspase-3/7 reagent according to manufacturer’s instructions and incubated for an additional 1.5 hours at room temperature. The fluorescence in each well was measured in triplicate (excitation at 485 nm, emission measured at 520 nm) using a microplate reader FLUOStar Omega (BMG-Labtech, Offenburg, Germany). For normalization of the data, the caspase activity in the control cells (exposed to 1% DMSO) was taken as 1.0. The apoptosis pathway was identified by measuring the activity of caspases 8 and 9 using Caspase-Glo 8 Assay and Caspase-Glo 9 Assay kits (Promega, Madison, WI, USA). After incubation of MOLT-4 and K562 cells with the test compounds for 3 h and 6 h, respectively, the cells were treated with the pro-luminescent substrates according to the manufacturer’s instructions and incubated for an additional 40 min at room temperature. Luminescence was measured (in triplicate) using a microplate reader FLUOStar Omega. For normalization of the data, the caspase activity in the control cells (exposed to 1% DMSO) was taken as 1.0.

### 2.4. Cleavage of Caspase 3/8/9 and Poly(ADP-Ribose)Polymerase (PARP)

0.15 × 10^6^ cells/ml were seeded on a 6-well plate (Nunclon, Thermo Fisher Scientific, Waltham, MA, USA). Following 24 hour preincubation the cells were treated with either the compound or 1% DMSO (control) for 24 or 48 hours. Cells were lysed using RIPA Buffer (Sigma) supplemented with Complete Protease Inhibitor Coctail (Roche, Basel, Switzerland) and PhosSTOP (Roche). Samples containing 20 µg protein were resuspended in 4× Laemmli’s Buffer (Biorad, Hercules, CA, USA), separated on 10% acrylamide gel (TGX Stain-Free Gel Kit, Biorad) and blotted onto nitrocellulose membrane (Panreac Applichem ITW, Darmstadt, Germany). Blots were blocked in 5% non-fat dry milk in TBST (1% (*v/v*) Tween-20, 140 mM NaCl, 25 mM Trizma, pH = 7.4 adjusted with HCl) for 1 h and incubated overnight with the antibodies against caspase-9 (9502), caspase-8 (9746), caspase-3 (9662), caspase-3 cleaved (9661) and β-actin (3700), all from Cell Signaling Technology, and antibody against poly(ADP-ribose)polymerase (PARP, 551025) from BD Pharmingen. Membranes were incubated with appropriate species-specific secondary antibodies conjugated with HRP (Cell Signaling Technology, Danvers, MA, USA). Blots were developed using ECL substrate (Biorad, Hercules, CA, USA). The experiment was performed in at least three independent biological replicates.

### 2.5. Annexin V/Propidium Iodide (PI) Flow Cytometry

The phosphatidylserine on the extracellular side of cell membranes was quantified using an FITC Annexin V Apoptosis Detection Kit I (BD Pharmingen, San Diego, CA, USA) according to the manufacturer’s instruction. 10^6^ cells were seeded on a 6-well plate (Nunc). 24 h later the cells were exposed to the test compounds at concentration of 5× IC_50_ for another 6 h (K562 cells) or 3 h (MOLT-4 cells). The control cells were exposed to 1% DMSO or 1μM staurosporine. After treatment the cells were centrifuged at 700 rpm (120× *g*) for 5 min, washed twice with cold PBS and then resuspended in 1x binding buffer to a density of 10^6^ cells/ml. 100 µL of the resuspended cells was stained in the dark with 5 μl of Annexin V-fluoroscein isothiocyanate (FITC) solution and 10 µL propidium iodide (PI) solution for 15 min at room temperature. The samples were then diluted with 400 μL of a binding buffer and within 1 h analyzed with a flow cytometer (BD FACSCalibur^TM^, BD Biosciences, San Jose, CA, USA). After treatment, four populations of cells were assessed: (1) viable (neither apoptotic nor necrotic, Annexin V-FITC and PI negative), (2) apoptotic (Annexin V-FITC positive and PI negative), (3) late apoptotic (Annexin V-FITC and PI positive), and (4) necrotic (Annexin V-FITC negative and PI positive).

### 2.6. Annexin V/7-Aminoactinomycin D (7-AAD) Flow Cytometry

0.15 × 10^6^ cells/ml were seeded on a 6-well plate (Nunclon, Thermo Fisher Scientific, Waltham, MA, USA). Following 24 h preincubation the cells were treated with either the compound or 1% DMSO (control) for 24 or 48 h. After indicated time, the cells were washed with cold PBS (w/o Mg^2+^, Ca^2+^), stained with Annexin V (FITC Annexin V Apoptosis Detection Kit with 7-aminoactinomycin D (7-AAD), Biolegend, San Diego, CA, USA) according to manufacturer instructions and analysed using BD FACSCalibur flow cytometer. Obtained Flow Cytometry Standard files were analyzed using BD FACSDiva Software (FACSDiva Version 6.1.3, Becton Dickinson Biosciences, San Jose, CA, USA). The experiment was performed three times in at least two technical replicates.

### 2.7. Real-Time Reverse Transcription Quantitative Polymerase Chain Reaction (RT-qPCR)

K562 tumor cells were seeded on a 6-well plate (Nunc, Roskilde, Denmark) in an amount of 3 × 10^6^ cells/well and were exposed to the test compounds at concentration of 5× IC_50_ for 18 h. The control cells were exposed to 1% DMSO or 1 μM staurosporine. The total RNA pool was isolated from the cells lysates using TriPure Isolation Reagent (Roche, Basel, Switzerland) according to the manufacturer’s instruction. Purity and integrity of RNA was checked spectrophotometrically with a NanoDrop ND-1000 spectrophotometer (Thermo Fisher Scientific, Waltham, MA, USA) and Agilent 2100 Bioanalyzer instrument. All RNA samples used for microarray studies had RIN values ≥ 9. Reverse transcription and polymerase chain reaction (RT-PCR) amplification reactions were performed in one step using a LightCycler® 1.0 Instrument (Roche) and LightCycler RNA Amplification Kit SYBR Green I (Roche). Each sample contained 250 ng of total RNA, 2 mM MgCl_2_, 2µl of 5× SYBR Green, 0.5 µM of forward and reverse primers, and 0.2 µl of the enzyme mix (total sample volume of 10 µl). The qRT-PCR reactions were optimized for each studied gene and performed according to a general protocol: a reverse transcription reaction (RT) for 10 min. at 55 °C, and a denaturation step for 30 s at 95 °C. The three steps of PCR quantification reaction included: I—denaturation (0 s at 95 °C), II—annealing (10 s at 60 °C), III—product extension (8 s at 72 °C), 45 cycles in total. Subsequent melting experiments were performed by quick denaturation at 95 °C, annealing over 10 s at 65 °C and heating up to 95 °C at 0.1 °C/s. The BAX, PMAIP1, HTRA2, TNFRSF 10B, and ESRRBL1 mRNA levels were normalized against a reference GAPDH mRNA. Changes in mRNA expression invoked by the tested compounds were calculated using ΔΔCt method (calibrator- RNA isolated with K562 cells exposed to 1% DMSO, averaged values ± standard deviation from 4 experiments are given). The following primers (sequences given in 5′ to 3′ direction) were used for PCR reactions: BAX Fwd: AAG GTG CCG GAA CTG ATC AG, Rev: GCG TCC CAA AGT AGG AGA GG, (product size 121 bp); PMAIP Fwd: GCT CAG GAA CCT GAC TGC AT, Rev: GCA CCC ATG AAT GCA CCT TC, (product size 138 bp); HTRA2 Fwd: TCC CTA TCT CGA ACG GCT CA, Rev: TGA ATC CTC AGC GTT GCG AT (product size 175 bp); TNFRSF10B Fwd: GAA GTT GGG CCT CAT GGA CA, Rev: GGT GTG GAC AGA GGC ATC TC (product size 127 bp); ESRRBL1 Fwd: CAG CTG AGT GAG GCA AAG GA, Rev: GGA GCA CCA TCA GTC ATG CT (product size 146 bp); GAPDH Fwd: CAT CAT CTC TGC CCC CTC TC, Rev: CTG TTG AAA CCA TAG CAC CT (product size 159 bp).

### 2.8. The Effect of Thalidomide, Lenalidomide and Test Dicarboximides on the IKZF1 and IKZF3 Level in Leukemia Cells

K562 or MOLT4 cells were seeded on 6-well plate (2 × 10^6^ cells/well) in a RPMI 1640 medium containing 10% FBS. MOLT4 cells were exposed to 1% DMSO (vehicle control), test compounds **6** (2 µM), **3** (5 µM), **5** (10 µM). Similarly, K562 cells were exposed to 1% DMSO, test compounds **6** (1 µM), **3** (1 µM), **5** (1 µM) for 48 h. Cells treated with 100 µM thalidomide or 10 µM lenalidomide served as positive controls. After incubation, cells were centrifuged (600 rpm, 6 min, 24 °C), washed once with PBS and lysed in 20 mM Tris (pH 7.2) containing 1% Triton X-100 supplemented with Complete® protease inhibitors cocktail (Roche). The levels of IKZF1 and IKZF3 were assessed by immunoblotting (IB).

### 2.9. IKZF1, IKZF3 and ABC50 Immunoblotting

Cells were lysed in TBS buffer containing 1% Triton X-100 and Complete® protease inhibitor cocktail (Roche) for 15 min on ice with occasional vortexing. Insoluble material was centrifuged (12,000 rpm, 15 min, 4 °C) and supernatants were collected. Total protein concentration was measured with DC Protein Assay (Bio-Rad, Hercules, CA, USA). Cell lysates containing 30 µg of total protein were mixed with Laemli buffer supplemented with SDS and β-mercaptoethanol, denatured (10 min, 95 °C) and subjected to sodium dodecyl sulfate polyacrylamide gel electrophoresis (SDS-PAGE) electrophoresis (4% stacking and 10% resolving gels). Resolved proteins were electrotransferred (semi-dry, 90 min, 1.5 mA/cm^2^) to a 0.45 µm nitrocellulose membrane (Thermo Scientific, Waltham, MA, USA). Membranes were blocked for 2 hours in TBST buffer (20 mM Tris-HCl pH 7.5, 0.9% NaCl, 0.1% Tween 20) containing 5% BSA (BioShop, Burlington, ON, Canada) and probed with primary antibodies (overnight at 4 °C) diluted in TBST buffer with 0.5% BSA. Tubulin served as a loading control for IB experiments. Anti IKZF3 (rabbit monoclonal, dilution 1:500), anti IKZF1 (rabbit polyclonal, dilution 1:500) were from Abcam. Anti IRF4 (rabbit polyclonal, dilution 1:500) were from Cell Signaling Technology. Anti α-tubulin (mouse monoclonal IgG, 1:1000) was from Sigma Aldrich. Anti ABC50 antibodies (mouse monoclonal ABC5H06, dilution 1:100) was from Abcam. After extensive washing with TBST buffer, membranes were probed with secondary goat anti-rabbit or goat anti-mouse HRP-conjugated IgG (1:5000 in TBST, Santa Cruz Biotech, Dallas, TX, USA) for 45 min at room temperature. Membranes were washed in TBST buffer and the chemiluminescent signal was developed with ECL Plus Western Blotting Substrate (Thermo Scientific) and visualized in Syngene G:Box detection system. Protein bands intensities were analysed using Image Quant (version 5.0, GE Healthcare Life Sciences, Pittsburgh, PA, USA).

### 2.10. Identification of Cellular Proteins Targeted by Dicarboximides—Pull-Down Assay

300 nmoles of biotinylated **6b** was added to 0.6 mL of the high capacity streptavidin agarose resin (Thermo Scientific, Waltham, MA, USA) equilibrated in 50% DMSO and incubated for 2 h (room temperature, gently mixing, total volume 0.7 mL). The K562 cell lysate was prepared in the buffer containing 0.5% NP-40 and supplemented with protease inhibitors coctail (Roche). Next, the beads with immobilized **6b** were added to the K562 cell lysate (9 mg of total protein) and incubated for 2 h at 4 °C with gently mixing. As a control, K562 cell lysate incubated with the streptavidin agarose beads was used. After incubation the beads were centrifuged (2000 rpm, 2 min, 4 °C) and washed 6 times with 5 mL of lysis buffer. After the last wash, the beads were resuspended in Laemli buffer and denatured 10 min at 95 °C. Released proteins (40 µL/well) were separated by SDS-PAGE (4% stacking, 8% resolving gel) and silver stained with Pierce Silver Stain Kit (Thermo Scientific, Waltham, MA, USA). Bands of interest were cut out of the gel and digested with trypsin. Tryptic peptides were separated by liquid chromatography (LC) and analyzed by LC-MS-MS/MS with Orbitrap mass spectrometer (Thermo). The sequences of the MS/MS spectra were identified by correlation with the human protein sequence database (Sprot 2016_11, H.) using MASCOT software (version 2.6.2, Matrix Science Inc., Boston, MA, USA) (http://www.matrixscience.com/). The significance threshold was *p* < 0.05 and ions score or expect cut-off was 22. 

### 2.11. Analysis of ABC50 Protein: Cell Culture, siRNA, Transfections

HeLa cells were transfected with a siRNA with the aid of Lipofectamine 3000 (Invitrogen, Carlsbas, CA, USA) according to manufacturer’s instructions. The complexes of Lipofectamine 3000 with RNA were prepared in Opti-MEM Reduced-Serum Medium (Gibco, Waltham, MA, USA). The ratio of Lipofectamine3000/nucleic acid (*v/w*) was 2/1. The final concentration of the siRNA was 100 nM for single siRNA transfections. Unless otherwise stated, cells transfected with a given siRNA were analyzed 48 h post-transfection. siRNAs for ABC50 (NM_001090) were designed with the aid of the siDESIGN Center (www.dharmacon.com), synthesized in-house (the Department of Bioorganic Chemistry, Centre of Molecular and Macromolecular Studies, Polish Academy of Sciences (CMMS PAS) and purified by high-performance liquid chromatography (HPLC). The silencing efficiency of siRNAs was determined by western blotting of ABC50 protein in transfected HeLa cells. The following siRNA duplexes (S—sense strand, AS—antisense strand) were used: 

si166 (S) 5′ CAG ACA AAG UGG UGA AGA ATT/(AS) 5′ UUC UUC ACC ACU UUG UCU GTT

si1612 (S) 5′ AUG ACC AGG GCU UCU UGG ATT/(AS) 5′ UCC AAG AAG CCC UGG UCA UTT

si1730 (S) 5′ AGA ACU GCU GAA ACA GUA UTT/(AS) 5′ AUA CUG UUU CAG CAG UUC UTT

Control siRNA (siCTL): (S) 5′ACA UGA AGC AGC ACG ACU UTT/(AS) 5′AAG UCG UGC UGC UUC AUG UTT

### 2.12. Statistical Analysis

Statistical analysis was performed with STATISTICA 10 (StatSoft, Round Rock, TX, USA) and Microsoft Excel (Excel 2010, Microsoft, Redmond, WA, USA). Routinely the t-test was used, while the non-parametric Mann–Whitney U test was applied for the data not fitting the normal distribution. P values below 0.05 were considered as statistically significant. For majority of presented experiments the results are given as a mean (± SD) from two or three independent experiments, each carried out at least in triplicate.

## 3. Results

### 3.1. Dicarboximides Are Cytotoxic toward Leukemia Cells

We have earlier shown [20] that dicarboximides **15** were highly cytotoxic towards human leukemia cells K562 and HL-60. Interestingly, these compounds were neither cytotoxic to adherent cancer cells (HeLa) nor to primary endothelial cells (HUVEC). Since K562 and HL-60 cells represent the chronic myeloid leukemia (CML) and acute myeloid leukemia (AML) type, respectively, we extended our studies to human MOLT-4 cells belonging to acute lymphoblastic leukemia (ALL). We also used the adherent human pancreatic cancer cell line CFPAC as the representative of the solid cancers. Please note, that in the biological tests described below, all dicarboximides were used as mixtures of diastereomers. Cytotoxicity of the test compounds **1**–**6** towards MOLT-4 and CFPAC cells was determined using the MTT test. Compounds **1**–**6** displayed significant cytotoxicity toward MOLT-4 and were non-toxic to adherent CFPAC cells, except of compound **6** (Table 2). These results support our previous observations that some dicarboximides demonstrate selective cytotoxicity against leukemia cells, particularly compounds **3** and **5** [20]. Compounds **3**, **5** and **6** were much more cytotoxic toward leukemia cells (IC_50_ values 1–20 µM) than cytarabine (IC_50_ 300 µM) and showed similar cytotoxicity as sorafenib and irinotecan (Table 2). Bortezomib and doxorubicin were highly cytotoxic to both cancer and normal cells. Although test dicarboximides were less potent than bortezomib or doxorubicin, their therapeutic indexes (Table 2) suggest that they might be safer in use, with less potential side effects. In this respect, test dicarboximides could be superior over standard anticancer drugs.

### 3.2. Dicarboximides Induce Apoptosis in Leukemia Cells

**Figure 1:**
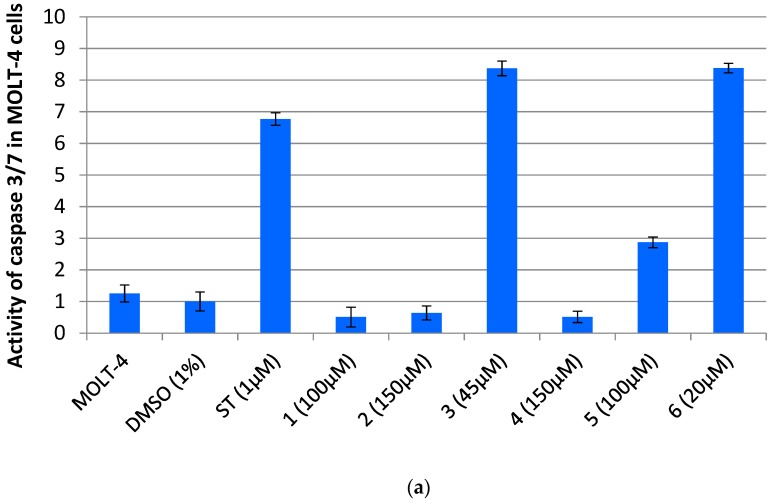

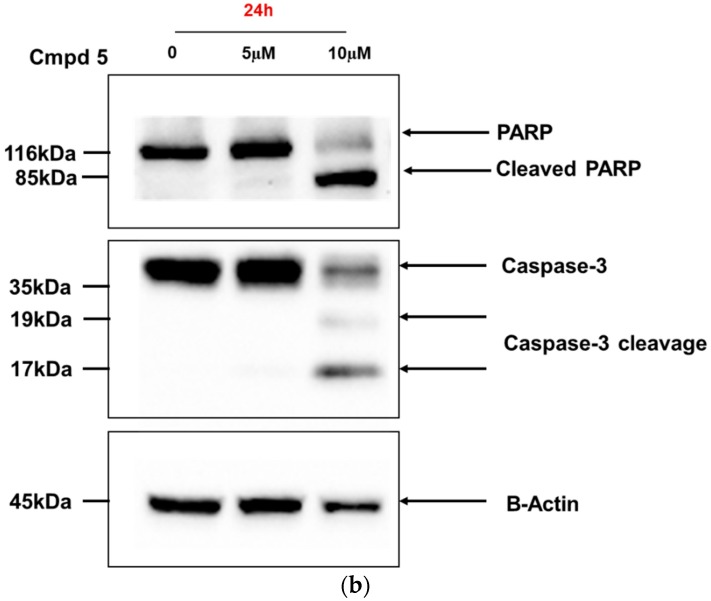
**Test dicarboximides activate caspase 3 and 7 in leukemia cells.** (**a**) Activity of caspase 3/7 in MOLT-4 cells treated with dicarboximides or staurosporine (positive control) for 18 h. The test compounds were used at the indicated concentrations (5× IC_50_). The results are normalized to the sample containing MOLT-4 cells exposed to 1% DMSO. Means ± SD are shown. Abbreviations: MOLT-4—untreated cells. ST—staurosporine. (**b**) Caspase 3 and poly(ADP-ribose)polymerase (PARP) cleavage in K562 cells treated with dicarboximide **5** for 24 h. Cells incubated with 1% DMSO were used as a control (0). After indicated time, lysates were collected and immunoblotted for caspase-3 and PARP cleavage. The experiment was performed in at least three independent biological replicates and representative Western blot results are shown.

We have previously reported that compounds **3** and **6** induce apoptosis in K562 (CML) leukemia cells [20]. Here we show that dicarboximides **1**–**6** are significantly cytotoxic for MOLT-4 (ALL) cells (Table 2). This finding prompted us to investigate further whether these compounds induce apoptosis in the MOLT-4 cells. In the below apoptosis studies (i.e., caspase 3/7/8/9 activation or cleavage assays, annexin V/PI staining) dicarboximides were used at the arbitrarily chosen concentration of 5× IC_50_. Apoptosis is characterized by strong activation of caspase 3 and 7 (caspase 3/7) which in turn cleave several key cellular proteins leading to cell death. Some of these cleaved proteins serve as markers of apoptosis, for example PARP. Another hallmark of apoptosis is a proteolysis of pro-caspase 3 yielding a 17 kDa protein fragment. Activity of caspase 3/7 can also be measured directly using profluorescent DEVD peptide substrate. As shown on Figure 1a, incubation of MOLT-4 cells with dicarboximides **3**, **5** and **6** led to a strong activation of caspase 3/7 suggesting that these compounds show a strong pro-apoptotic activity. Using different assays, we demonstrated that compound **5** induced apoptosis in K562 cells. This is evidenced by the presence of cleavage fragments of PARP (85 kDa) and caspase 3 (17 kDa) (Figure 1b).

**Figure 2:**
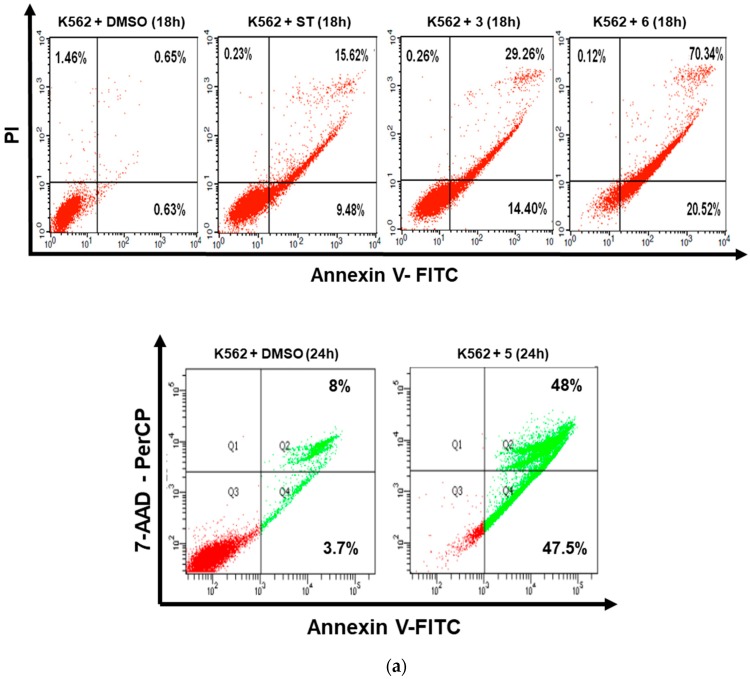

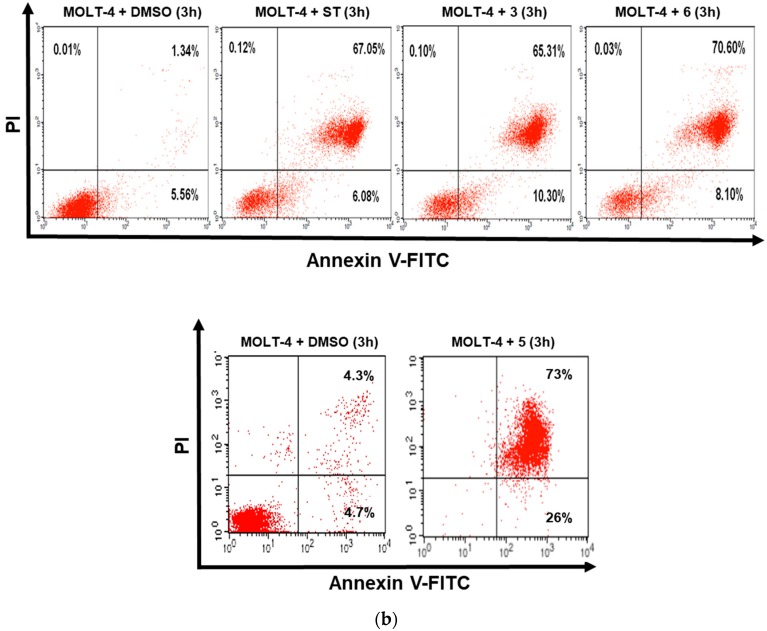
Flow cytometry analysis of apoptosis in leukemia cells induced by the test compounds or staurosporine. Apoptosis was determined by Annexin V Fluoroscein Isothiocyanate (FITC) Apoptosis Detection Kit I, (BD Pharmingen). The test compound and time of incubation with cells is given above each scatterplot. (**a**) scatterplots of K562 cells stained with Annexin V FITC and PI (or 7-AAD, FITC Annexin V Apoptosis Detection Kit with 7-AAD, BioLegend). Abbreviations: K562 + DMSO—cells treated with 1% DMSO; ST—staurosporine. Tested compounds were used at the following concentrations: **3** (10 µM), **6** (5 µM), **5** (5 µM); staurosporine was used at 1µM concentration; (**b**) scatterplots of MOLT-4 cells stained with Annexin V FITC and PI. Abbreviations: MOLT-4 + DMSO—cells treated with 1% DMSO. Tested compounds were used at the following concentrations: **3** (45 µM), **6** (20 µM), **5** (100 µM).

To confirm the ability of compounds **3**, **5** and **6** to induce apoptosis in leukemia cells, we tested phosphatidylserine (PS) distribution in K562 and MOLT-4 cells using fluorescently labelled annexin V. In the non-apoptotic cells, PS is present only in the inner (cytoplasmic) leaflet of the cell membrane. During apoptosis PS undergoes translocation to the outer leaflet giving a signal for phagocytosis. The cells were treated with **3**, **5** and **6** (at the concentrations of 5× IC_50_) or 1 µM staurosporine (positive control) and 1% DMSO (negative control). Necrotic cells were visualized with propidium iodide (PI) or 7-aminoactinomycin D (7-AAD). As shown in Figure 2a, dicarboximides **3**, **5** and **6** induced apoptosis in K562 cells (please note the different incubation times for compounds **3**, **6** and **5**). Staurosporine (positive control) induced apoptosis in only about 25% of K562 cells including both early (LR) and late (UR) apoptotic cells. In the presence of **5** and **6** we observed more than 90% of apoptotic cells, while compound **3** induced apoptosis in about 44% of K562 cells. Similar results were obtained in MOLT-4 cells. In the preliminary experiments we found that MOLT-4 cells were more sensitive to the apoptosis-inducing factors therefore, only 3h incubation with staurosporine or test compounds was applied. The results shown in Figure 2b indicate that compounds **3**, **5** and **6** induced massive apoptosis in about 76%, 78% and 99% of MOLT-4 cells, respectively and the late apoptotic phase was prevailing. Altogether, these data further confirm that compounds **3**, **5** and **6** induce efficient and extensive apoptosis in K562 and MOLT-4 cells.

### 3.3. Dicarboximides Activate Apoptosis via Receptor and Mitochondrial Pathways

**Figure 3:**
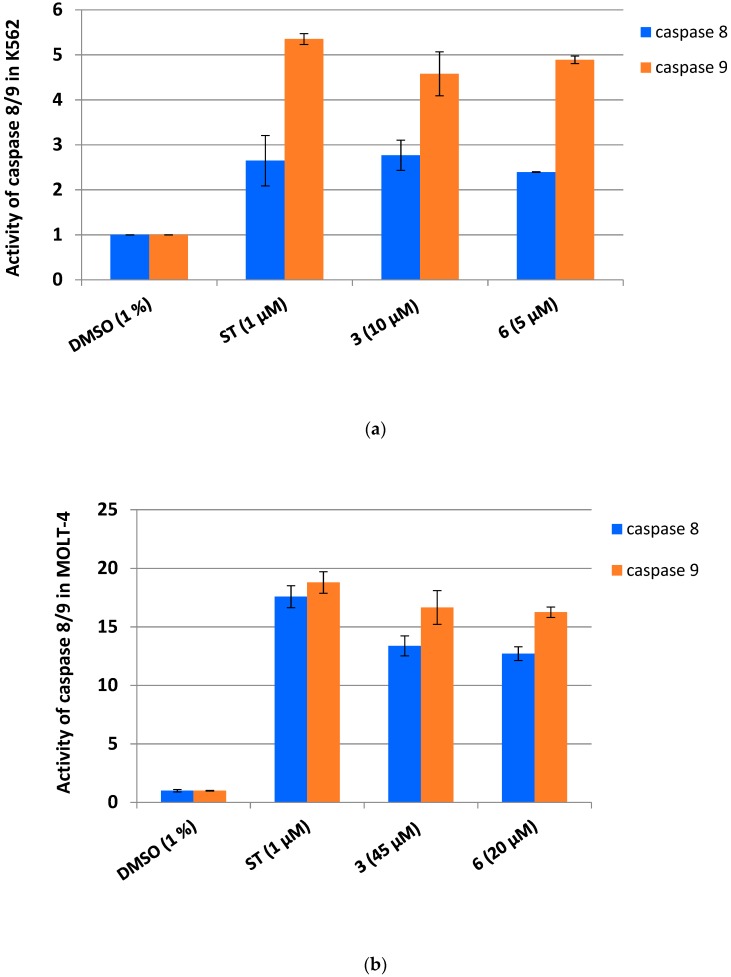

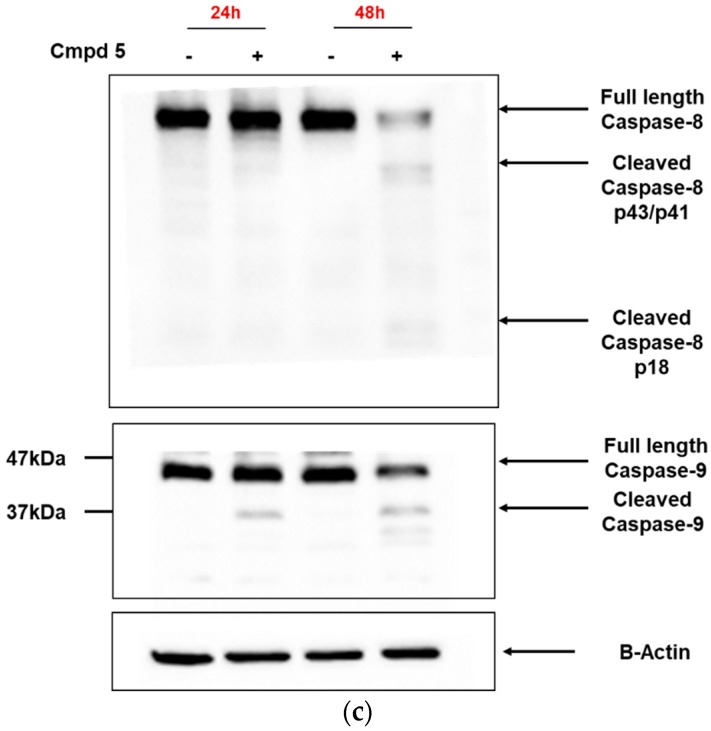
Activation of caspase 8 and caspase 9 in leukemia cells treated with compounds **3**, **5** and **6**. K562 (**a**) or MOLT-4 cells (**b**) were treated with **3**, **6** or staurosporine (ST) for 6 or 3 hours, respectively. Activity of caspases in cells exposed to 1% DMSO was normalized to 1.0. Means +/− SD are shown. (**c**) Western blot analysis of cleavage of caspase 8 and caspase 9 in K562 cells incubated with **5** (5 µM) for 24 and 48 hours. Cells incubated with 1% DMSO were used as a control. After indicated time, lysates were collected and immunoblotted for cleavage of caspase 8 and caspase 9. The experiment was performed in at least three independent biological replicates and representative Western blot results are shown.

To identify which of the apoptotic pathways: receptor or mitochondrial is activated by dicarboximides, we measured the activity of caspase 8 and 9 (caspases 8/9) in leukemia cells exposed to compounds **3**, **5** and **6**. In non-apoptotic cells, caspases 8/9 exist as inactive pro-enzymes which are cleaved upon initiation of apoptosis, yielding enzymatically active caspases. Activated (cleaved) caspases 8 and 9 are markers of the receptor and mitochondrial apoptotic pathway, respectively. Figure 3 clearly indicates that caspases 8/9 were strongly activated in K562 treated with **3**, **5**, **6** and in MOLT-4 cells treated with **3** and **6**. Treatment of K562 cells with **3** or **6** resulted in **a** more than 2-fold increase of activity of caspases 8/9 compared to the control (Figure 3a). Even stronger activation of caspases 8/9 (more than 10-fold) was observed in MOLT-4 cells exposed to **3** or **6** (Figure 3b). Incubation of K562 cells with compound **5** also led to the activation of caspases 8/9. Cleaved (i.e., activated) forms of caspases 8/9 were detected in K562 cells after 48 h incubation with **5**, while after 24 h we observed cleaved form of caspase 9 (Figure 3c). These results indicate that compounds **3**, **5** and **6** induce apoptosis in leukemia cells by both the receptor and mitochondrial pathways.

### 3.4. Dicarboximides Change the Expression Profile of Genes Involved in Apoptosis 

**Figure 4:**
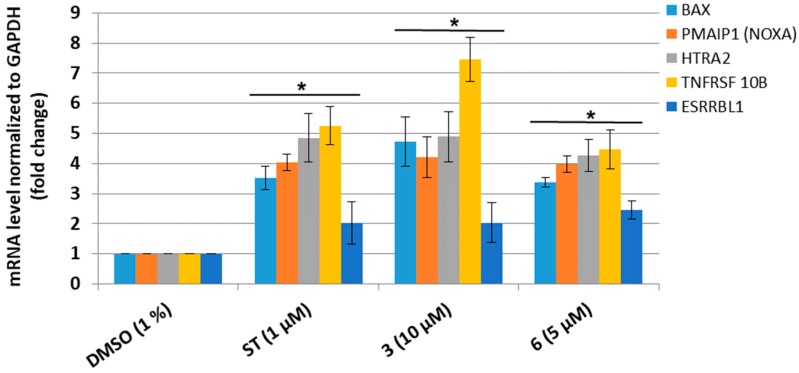
Real time reverse transcription quantitative polymerase chain reaction (RT-qPCR) analysis of relative mRNAs levels in K562 cells after 18 h of incubation with staurosporine (1 µM, positive control) and tested compounds **3** (10 µM) and **6** (5 µM). Levels of analyzed mRNAs in K562 cells treated with 1% DMSO (reference sample) were expressed as 1.0. Statistically significant changes are indicated with asterisks (*p* < 0.05, Mann-Whitney U test).

Furthermore, we assessed the expression of five apoptosis-related genes using real-time RT-qPCR. The selected genes encode the following proteins: *BAX* (BCL-2-associated X protein), *PMAIP1 (NOXA)* (phorbol-12-myristate-13-acetate-induced protein 1), *HTRA2 (OMI)* (high temperature requirement A2 serine protease), *TNFRSF 10B (DR5)* (tumor necrosis factor receptor superfamily, member 10B), and *ESRRBL1 (IFT57)* (intraflagellar transport 57 homolog (Chlamydomonas)). As shown in Figure 4 dicarboximides **3** and **6** significantly induced the expression of mRNAs encoding proteins involved in both the receptor (TNFRSF 10B, ESRRBL1) and mitochondrial (BAX, PMAIP1, HTRA2) apoptotic pathways. The concentration of dicarboximides was kept the same as in the apoptosis activation studies i.e., 5× IC_50_. The expression profiles of analyzed mRNAs obtained upon the use of **3** and **6** were similar to staurosporine. These data corroborate with our results demonstrating that test dicarboximides activated caspase 8/9 leading to apoptosis via receptor and mitochondrial pathways.

### 3.5. Identification of Cellular Proteins Targeted by Dicarboximides—Pull-Down Assay 

**Figure 5:**
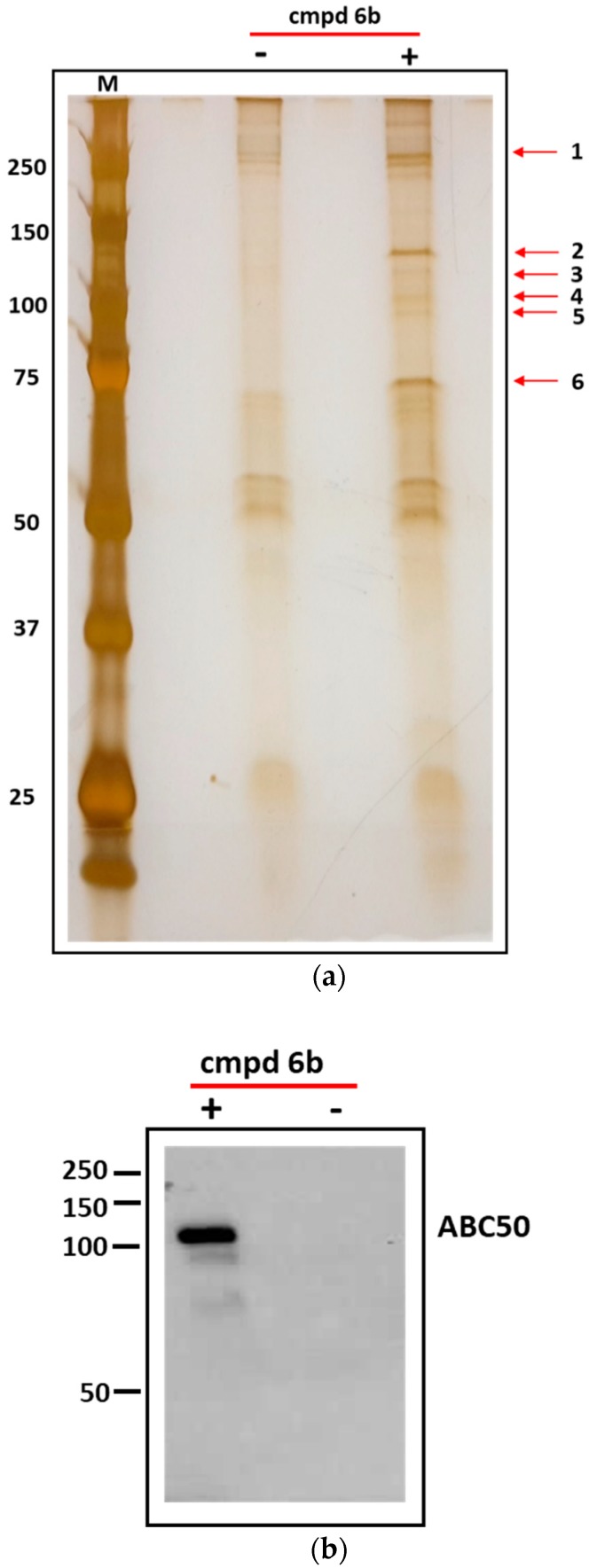
(**a**) SDS-PAGE of the streptavidin-agarose pull down samples obtained from lysates of K562 alone or incubated with **6b**. The gel was silver stained. M—protein marker lane in kDa. Numbers on the right correspond to band number entries in Table 3 (below). (**b**) Western blot identification of ABC50 in a material pulled-down from lysates of K562 cells. Mouse monoclonal anti ABC50 antibodies were used. Pull-down was performed in the presence (+) or in the absence (−) of compound **6b**.

To identify cellular targets for the test dicarboximides, we have synthesized a biotinylated derivative of **6a**. Compound **6a** exhibited significant cytotoxicity toward leukemia cells and was chosen for biotinylation due to the presence of hydroxyl and amine groups (Table 2). The biotinylated derivative **6b** retained cytotoxicity toward K562 and MOLT-4 cells with IC_50_ of 7 and 1.5 µM, respectively (Table 2). Next, **6b** was immobilized on streptavidin-agarose beads and incubated with the lysate of K562 cells. Bound cellular proteins were pulled-down, separated by SDS-PAGE and visualized by silver staining. We have observed six protein bands located between 250 and 75 kDa that were uniquely present or significantly enhanced in a pull-down material with **6b** (Figure 5a). These bands were excised, and their protein content was identified by mass spectrometry (Supplementary materials). In five out of six bands analyzed, the ABC50 protein was identified and significantly enriched (Table 3). Western blot analysis of pull-down material with immobilized **6b** confirmed the presence of ABC50 (Figure 5b). Therefore, we focused on the ABC50 protein as a target for the test dicarboximides.

### 3.6. ABC50 Knockdown Abolishes HeLa Sensitivity to Dicarboximide **6**

**Figure 6:**
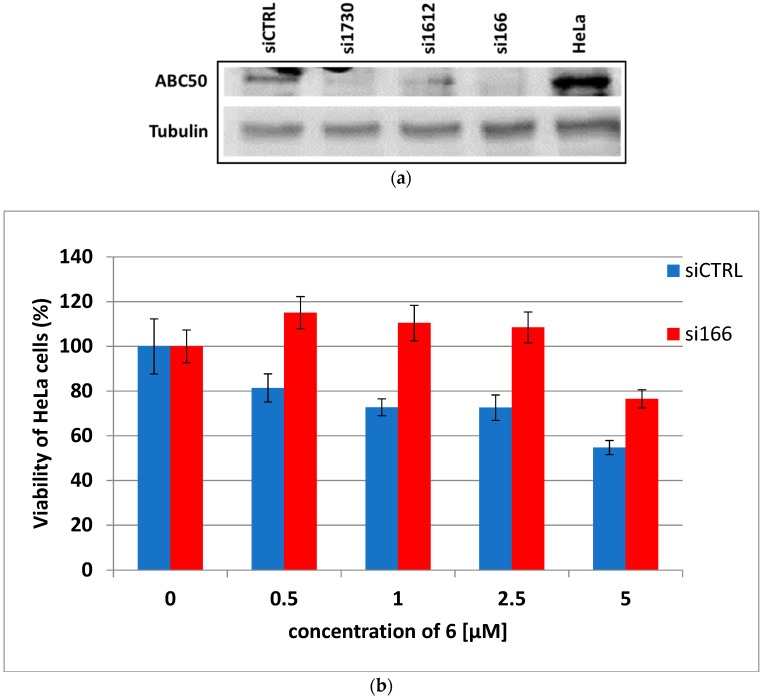
(**a**) Downregulation of ABC50 by siRNA. HeLa cells were transfected with a 100 nM of siRNA. 48 h posttransfection ABC50 levels were determined by Western blotting. ABC50 level in cells transfected with control siRNA (siCTRL) was used as a reference. Tubulin served as a loading control. HeLa—non-transfected cells. (**b**) siRNA-mediated knockdown of ABC50 impairs cytotoxicity of **6** in HeLa cells. Cells were transfected with the indicated siRNAs and treated with increased concentrations of **6**. Cells incubated with 1% DMSO were used as a control (0).

Results of the pull-down experiments strongly suggested that ABC50 might be a cellular target for test dicarboximides. To confirm the role of this protein in dicarboximides-induced cytotoxicity, we investigated whether siRNA-mediated downregulation of ABC50 renders the cells less sensitive to **6.** First, we tried to deliver siRNA to K562 or MOLT-4 cells either by transfection or nucleofection (electroporation). Despite several attempts, we were not able to efficiently downregulate the expression of ABC50 in K562 or MOLT-4 cells, probably due to inefficient delivery of siRNA. Therefore, we switched to HeLa cells which are more prone to transfection and they are also sensitive to compound **6** (Table 2). We identified siRNA designated as si166, that efficiently downregulated ABC50 protein in HeLa cells (Figure 6a). Subsequently, Hela cells were transfected with si166 and 24h later were treated with increased concentrations of **6** (i.e., 0.5, 1, 2.5 and 5× IC_50_). After additional 48 h the viability of cells was measured with an MTT assay. As shown in Figure 6b, HeLa cells with downregulated ABC50 were less sensitive to **6** when compared with control cells, i.e., transfected with control siRNA (siCTL). In the presence of **6** at the concentration of up to 2.5 µM the viability of HeLa cells with knocked-down ABC50 (HeLa kdABC) was not affected, while the viability of cells with normal levels of ABC50 was significantly lower. Only at the highest concentration of **6** (i.e., 5 µM) the viability of HeLa kdABC was reduced about 20%. These data confirm that observed cytotoxicity of dicarboximides is likely the result of targeting the ABC50 protein.

### 3.7. Dicarboximides Reduce the Level of IKZF1/3 Transcription Factors in Leukemia Cells

**Figure 7:**
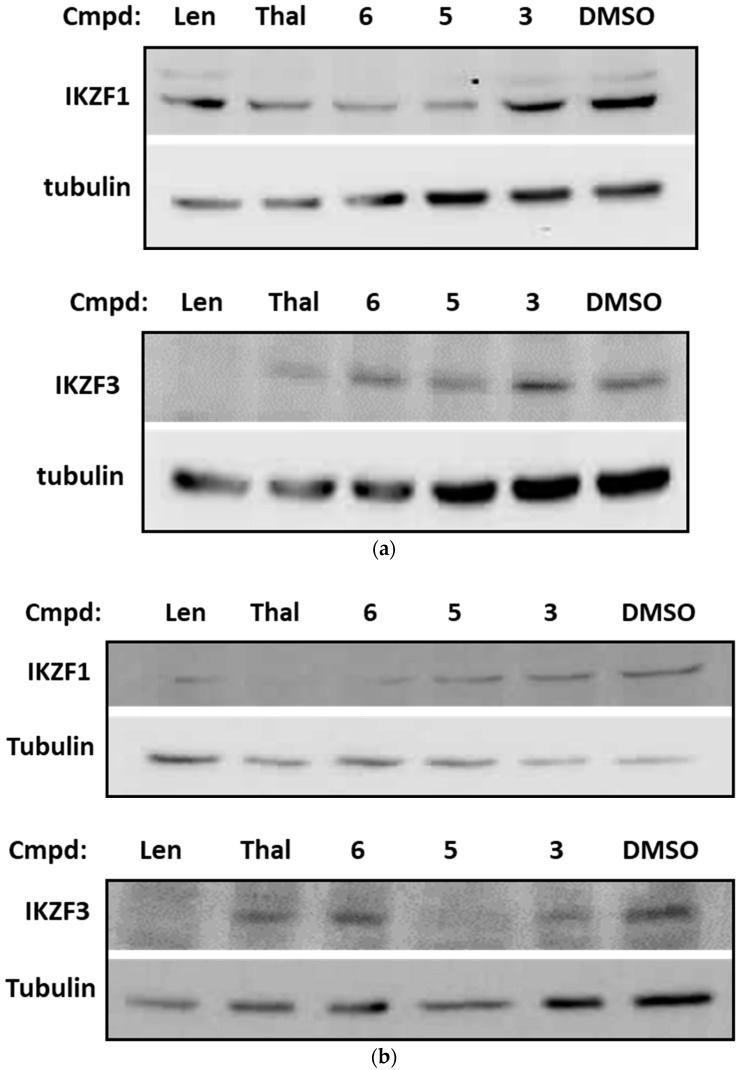
Dicarboximides downregulate the levels of IKZF1 and IKZF3 transcription factors in leukemia cells. (**a**) K562 cells treated with lenalidomide (10 µM), thalidomide (100 µM) and dicarboximides **3** (1 µM), **5** (1 µM) or **6** (1 µM) for 48 h. (**b**) MOLT4 cells treated with lenalidomide (10 µM), thalidomide (100 µM) and dicarboximides **3** (5 µM), **5** (10 µM) or **6** (2 µM) for 48 h. Representative blots from at least 3 independent experiments are shown.

Thalidomide and its derivatives (lenalidomide and pomalidomide) belong to the class of organic compounds known as phthalimides and display immunosuppressive and anti-angiogenic activity. Thalidomide binds to the cereblon protein and modifies the function of the E3 ubiquitin ligase leading to downregulation of the transcription factors: Ikaros family zinc finger protein-1 (IKZF1, Ikaros) and 3 (IKZF3, Aiolos). Similarities in chemical structure between the test dicarboximides and thalidomide, i.e., the presence of phthalimide-like moiety, allowed us to hypothesize that the mechanism of action might be similar. We examined whether incubation of K562 and MOLT-4 cells with **3**, **5** and **6** had any effect on the level of IKZF1 and IKZF3. Dicarboximides were evaluated at the concentrations of IC_50_ (K562) or 0.5× IC_50_ (MOLT-4), to limit the cytotoxic effect. As shown in Figure 7, we observed a significant diminution of IKZF1 and IKZF3 in the leukemia cells treated with compounds **5** and **6**. This effect was similar to lenalidomide or thalidomide used as reference compounds. Incubation of cells with compound **3** led to a modest reduction of IKZF1 and IKZF3. These results support our hypothesis that the biological activity of the test dicarboximides is similar to thalidomide, i.e., they modulate the level of IKZF1 and IKZF3 in leukemia cells.

### 3.8. Glutarimide Derivatives of Dicarboximides as Modulators of ABC50 in Cancer Cells—Proteolysis-Targeting Chimeras (PROTACs) Approach

**Figure 8:**
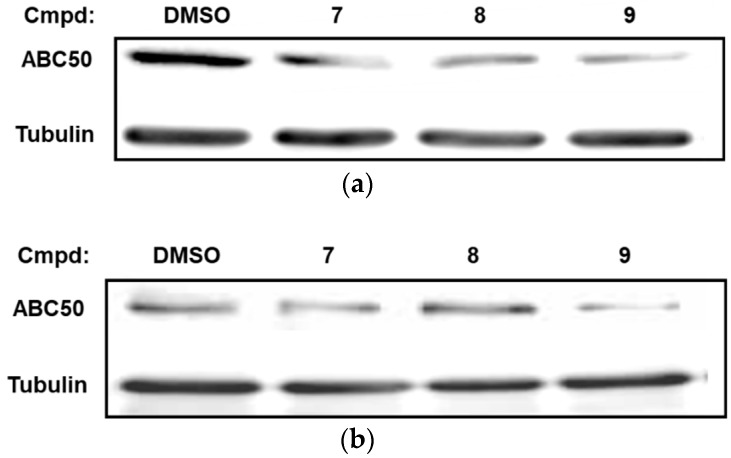
The effect of dicarboximide-based proteolysis-targeting chimeras (PROTACs) on the ABC50 protein in the (**a**) K562 or (**b**) HeLa cells. Cells were incubated with compounds for 96 h and the ABC50 was detected by Western blotting. Compounds **7** and **9** were used at 30 µM, **8** was used at 15 µM, DMSO 1% (control). Representative blots from at least 3 independent experiments are shown.

ABC50 was identified as the target of the test dicarboximides in the pull-down experiments. Therefore, we examined whether dicarboximides could be used as proteolysis-targeting chimeras (PROTACs) to downregulate the ABC50 protein in leukemia cells. We synthesized the chimeras of **3**, **5** and **6** in which alkylamine chain was replaced with glutarimide, while the phthalimide-like portion remained unchanged or slightly modified (as in the case of **6**, where the methylene bridge in the phthalimide-like ring was replaced by ketone bridge in **9**). These chimeras are shown in Table 1 as **7**, **8** and **9**. A glutarimid ring endows the chimeras with the structural basis to bind cereblon protein, a part of E3 ubiquitin ligase complex. The phthalimide-like portion of chimeras would bind ABC50 protein thus facilitating its ubiquitination by E3 ubiquitin ligase and subsequent degradation in a proteasome. It must be noted that **7**, **8** and **9** were evaluated as mixtures of enantiomers. K562 and HeLa cells were incubated with chimeras **7** (30 µM), **8** (15 µM) and **9** (30 µM) for 96 h and subsequently the level of ABC50 was determined by Western blotting. The concentrations of **7**, **8** and **9** were below their IC_50_ to minimize the cytotoxic effect. Figure 8a shows the level of ABC50 was significantly reduced by chimeras **7**, **8** and **9** in K562 cells. A weaker anti-ABC50 effect was observed in HeLa cells (Figure 8b). Compounds **7** and **8** significantly downregulated ABC50, while **9** didn’t have such pronounced effect. These results demonstrate that test dicarboximides may be used as PROTACs for the inhibition of ABC50 in cancer cells.

## 4. Discussion

We have earlier shown [20] that the compounds **1**–**6** (enlisted in Table 1) were selectively cytotoxic towards adherent (HeLa) or suspension (K562, HL-60) cancer cells, being non-toxic towards normal HUVEC cells. In the present studies we aimed to characterize the pathway of a cell death (necrosis vs apoptosis) and to identify mechanism of activity and cellular protein target(s) of the test dicarboximides. Due to structural similarities between test dicarboximides and thalidomide (presence of the phthalimide-like ring), we also evaluated their immunomodulatory activity, i.e., the effect on IKZF1 and IKZF3.

We determined cytotoxicity of the screened compounds towards two additional cancer cell lines, pancreatic cancer CFPAC and leukemia MOLT-4. The corresponding IC_50_ values were in the range of 1.5–40 µM (Table 2). Interestingly, compounds **1**–**6** were not cytotoxic towards normal HUVEC cells, and except for **6**, towards adherent HeLa and CFPAC cells. This indicates that the test dicarboximides show, to some degree, selective cytotoxicity for leukemia cells. We also compared the cytotoxicity of the screened dicarboximides with reference drugs: cytarabine, bortezomib and doxorubicin (anti-leukemia drugs), sorafenib (used in the therapy of liver and kidney cancer) and irinotecan (colon cancer). The results summarized in Table 2 indicate that the IC_50_ values of dicarboximides in the leukemia cells are significantly lower than cytarabine and similar to sorafenib or irinotecan. However, it must be emphasized that the test compounds are virtually non-toxic towards primary endothelial cells, while the reference drugs (except for cytarabine) show potent cytotoxicity against these cells. Therefore, the test dicarboximides might be clinically more beneficial than the standard anticancer drugs.

Cytotoxicity of **3**, **5** and **6** towards K562 and MOLT-4 cells correlated with increased activity of caspases 3 and 7 (Figure 1) and enhanced Annexin-V staining (Figure 2). Thus, **3**, **5** and **6** exert cytotoxicity towards K562 and MOLT-4 cells by inducing massive and effective apoptosis. Moreover, these compounds increased the activity of caspases 8 and 9 (Figure 3) in K562 and MOLT-4 cells, indicating activation of both, receptor and mitochondrial apoptotic pathways. This conclusion is further supported by the results of expression of several pro-apoptotic genes. We have found that **3** and **6** significantly upregulated the expression of genes involved in the receptor (*TNFRSF 10B, ESRRBL1*) and in the mitochondrial (*BAX, PMAIP1, HTRA2*) pathways of apoptosis (Figure 4). In this respect, the test dicarboximides resemble other derivatives, for example, naphthalimides and maleimides. It was demonstrated that naphthalimides and maleimides activated the mitochondrial apoptotic pathway by changing the mitochondrial potential, increasing the level of the AIF and pro-apoptotic Bax proteins, and decreasing the level of anti-apoptotic proteins Bcl-2 and survivin [10,24]. On the other hand, maleimides activated the receptor apoptotic pathway by increasing the level of the membrane Fas receptor in Jurkat cells [25]. The profiles of gene expression (Figure 4) were similar to control experiment, where the cells were treated with staurosporine. Staurosporine is mainly involved in the activation of mitochondrial pathway of apoptosis, but the receptor and the caspase-independent pathways are not excluded [26,27].

In a pull-down assay followed by mass spectrometry analysis, ABC50 (or ABCF1) protein was identified as a possible target of the test dicarboximides. For this experiment we used biotinylated derivative **6b**. Its cytotoxic properties were slightly diminished comparing the parent **6a**. Biotinylation may lead to a different pattern of hydrogen bonds the compound can form with biomolecules. The reduced selectivity of **6b** toward K562 cancer cells and increased trifold IC_50_ for MOLT-4 cells can result from different steric hindrance of hydroxyl group and/or chemical “inactivation” (in terms of hydrogen bonding) of the amine (-NH_2_) group when compared to the parental compound **6a**. In addition, compound **6b** may also interfere with important metabolic processes that depend on biotin, a coenzyme for carboxylases. We also demonstrated that HeLa cells with knocked-down ABC50 showed increased resistance to the test dicarboximides, confirming the ABC50 as a target. ABC50 belongs to a family of ATP-binding cassette (ABC) proteins. Most ABC proteins are membrane-bound ATP-dependent pumps. In contrast, ABC50 lacks a transmembrane domain and does not function as a transmembrane transporter. This protein locates primarily in the cytoplasm and nucleoplasm of cells, where it binds to eukaryotic initiation factor 2 (eiF2) and promotes interaction between eiF2 and methionyl-tRNA. This suggests that ABC50 is involved in ribosome biogenesis [28] and initiation and control of protein synthesis [29]. Lindqvist et al. demonstrated that inhibitors of protein synthesis trigger the cell death [30]. It was also demonstrated that siRNA-driven depletion of ABC50 in HeLa cells led to the inhibition of total protein synthesis [29]. Thus, the observed cytotoxicity of dicarboximides and their pro-apoptotic activity could result from binding to ABC50 and impairing protein synthesis. Other studies indicate that ABC50 is an important regulator of innate immune responses and autoimmune disorders. Lee et al. demonstrated that si-RNA mediated knockdown of ABC50 in mouse embryonic fibroblasts (MEFs) resulted in a significant inhibition of synthesis of CXCL10 in response to interferon-stimulatory DNA [31]. Other study indicated that TNF-alpha strongly increased the expression of ABC50 mRNA in human synoviocytes [32]. Increased copy number of *ABC50* gene was also associated with increased risk of gout and autoimmune pancreatitis [33,34].

Having demonstrated that the test dicarboximides target ABC50 and based on their structural similarity to IMIDs (i.e., thalidomide, lenalidomide), we hypothesized that dicarboximides might inhibit the synthesis and/or induce proteasome-dependent degradation of immunomodulatory proteins. Indeed, incubation of leukemia cells with the test dicarboximides resulted in downregulation of transcription factors IKZF1 and IKZF3 (Figure 7), which are particularly important for development of T- and B-lymphocytes. Among dicarboximides, compounds **1**–**5** differ in an amino alkyl substituent attached to the imide nitrogen atom. Unfortunately, we are unable to provide any rationale for changes in their activity (especially in HL60 cells) upon change of that substituent. Although from the chemical point of view, these differences are only minute, one cannot exclude that they are important in highly specific interactions, which to us remain obscure. Compounds **3**, **5** and **6** show the most potent cytotoxicity. While compounds **3** and **5** possess the same phthalimide-like moiety as compounds **1**, **2** and **4**, their amino alkyl/hydroxyl-amino alkyl chains are the most extended. Compound **3** contains ethyl chain linked to piperidine residue via the nitrogen atom, while **5** has an n-propanol chain linked to isopropyl amine (secondary amine), and such a structure offers both—hydrophobic and hydrogen bond interactions with the target biomolecule. On the other hand, compound **6** has the most extended phthalimide-like part, with the central ring substituted with 4 phenyl groups and with the methylene bridge. Compound **6** exhibits higher cytotoxicity than **1**, while both compounds have the same amino alkyl chain but differ in phthalimide-like portion, so, in principle, it might be possible than this “extended” aromatic system may more efficiently interact with the target biomolecules by stacking interactions or exert beneficial intercalating properties. Unfortunately, hydrolysis of plasmid DNA with restriction endonuclease provided no evidence that such a process operates to any measurable extent [20].

Moreover, we obtained novel PROTAC chimeras by extending the dicarboximides **3**, **5** and **6** with a glutarimide fragment of thalidomide. They target the ABC50 protein via the dicarboximide moiety and recruit the E3 ubiquitin ligase by the glutarimide part. In cellular experiments, they proved to be effective tools for presumably proteasome-dependent downregulation of ABC50 protein in cells.

Based on the obtained results we propose the putative mechanisms of action of dicarboximides **1**–**6** and **7**–**9**, which are schematically depicted below. 
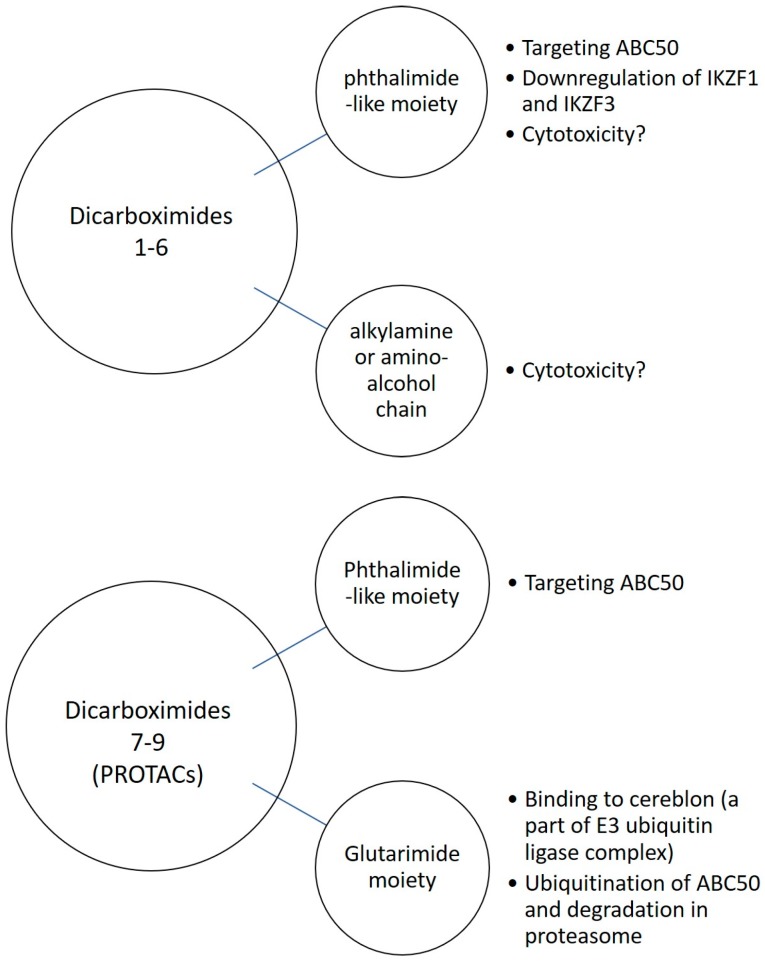


## 5. Conclusions

In summary, we have identified novel dicarboximides that are cytotoxic for cancer cells and show some preference toward leukemia cells. We also provided evidence that these compounds induced leukemia cells death by activating receptor and mitochondrial apoptotic pathways. The molecular mechanism of their cytotoxicity depends at least in part on ABC50 protein, which was identified as a target of dicarboximides in a pull-down assay. We also demonstrated that dicarboximides could be successfully used as tools for targeted proteolysis of ABC50. Similar to thalidomide, the test dicarboximides displayed immunomodulatory activity and decreased the levels of IKZF1 and IKZF3 transcription factors. Thus, biological activity of the test dicarboximides makes them potential candidates for being lead compounds for synthesis and biological evaluation of new anticancer agents.

## 6. Patents

The compounds described in this publication and their application are the subject of European and Polish Patent Applications: EP13176421.9, EP13150611.5, P.400000, P.398193.

## Figures and Tables

**Table 1:** Chemical structure and the numbering of dicarboximides.

Compound	Structure *	Compound	Structure *
**1**	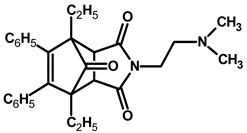	**4**	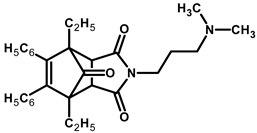
**2**	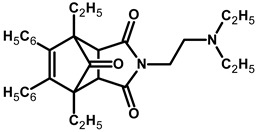	**5**	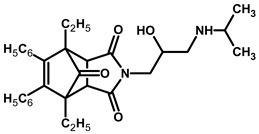
**3**	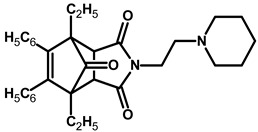	**6**	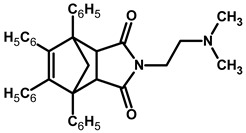
**6a**	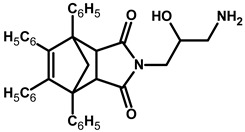	**6b**	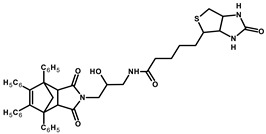
**7**	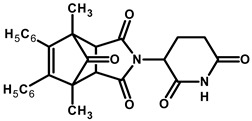	**8**	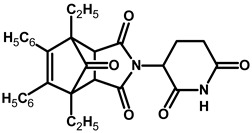
**9**	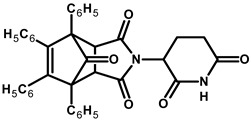		

* all amine derivatives of dicarboximides were used as hydrochlorides.

**Table 2:** Cytotoxicity of test compounds, cytarabine, bortezomib, sorafenib, irinotecan and doxorubicin in different cell lines after 48 h. IC_50_ mean values [µM] ± standard deviation (SD) are shown.

Compound	HeLa	K562	HL-60	HUVEC	MOLT-4	CFPAC	TI ***
cytarabine	>1000 *	300 ± 19 *	300 ± 18 *	>1000 *	nd	nd	3.3
bortezomib	0.05 ± 0.01 *	0.04 ± 0.01 *	0.3 ± 0.1 *	<0.01 *	nd	nd	0.25
sorafenib	20.0 ± 2.6 *	3.0 ± 0.3 *	15.0 ± 0.6 *	20.0 ± 4.1 *	nd	nd	6.7
irinotecan	200 ± 11 *	10.0 ± 0.9 *	70.0 ± 2.5 *	30.0 ± 3.7 *	nd	nd	3
doxorubicin	0.50 ± 0.01 *	0.15 ± 0.01 *	0.15 ± 0.03 *	0.10 ± 0.03 *	nd	nd	0.67
**1**	>100 **	10 ± 0.59 **	>100 **	>100 **	20.0 ± 2.7	>1000	10
**2**	>100 **	4.5 ± 0.29 **	>100 **	>100 **	30.0 ± 2.3	>1000	22
**3**	>100 **	2.0 ± 0.31 **	>100 **	>100 **	9.0 ± 0.6	>1000	50
**4**	>100 **	8 ± 0.53 **	>100 **	>100 **	30.0 ± 2.0	>1000	12.5
**5**	>100 **	1.0 ± 0.05 **	2.0 ± 0.05 **	>100 **	20.0 ± 2.3	>1000	100
**6**	1.0 ± 0.2 **	1.0 ± 0.3 **	5.0 ± 0.24 **	>100 **	4.0 ± 0.2	40.0 ± 3.1	100
**6a**	20 ± 1.9	12 ± 1.5	nd	>100	5 ± 0.59	nd	83
**6b**	64 ± 10	7 ± 1.2	nd	3.8 ± 0.22	1.5 ± 0.06	nd	0.5
**7**	180	170	nd	200	150	nd	1.2
**8**	>200	25	nd	65	18	nd	2.6
**9**	>200	>200	nd	>200	>200	nd	nd

nd—not determined, * taken from [23], ** taken from [20], *** TI was calculated as a ratio of IC_50_ (concentration required to reduce cell survival fraction to 50% of control) for HUVEC and K562.

**Table 3:** Identification of cellular proteins pulled-down with **6b**.

Band No.	Database	Entry	Score	Mass (Da)	No. of Significant Matches	No. of Significant Sequences	Exponentially Modified Protein Abundance Index (emPAI)	Description
1	Sprot	Q9Y490	2693	271,766	72	59	18,629	Talin-1
2	Sprot	Q8NE71	289	96,323	8	8	0.42	ATP-binding cassette sub-family F member 1 (ABCF1 or ABC50)
3	Sprot	Q8NE71	565	96,323	15	14	0.85	ABC50
4	Sprot	Q8NE71	1923	96,323	43	29	32,905	ABC50
5	Sprot	Q8NE71	651	96,323	18	16	42,767	ABC50
6	Sprot	Q8NE71	495	96,323	12	11	0.62	ABC50

## Supplementary Materials

The following are available online at https://www.mdpi.com/2218-273X/9/9/446/s1, Figure S1: The numbering of atoms of derivative **6b** used in the description of nuclear magnetic spectra, Figure S2: The numbering of atoms of compounds **7**–**9** used in the description of nuclear magnetic spectra, Table S1: Tests and cell lines used to characterize the biological activity of dicarboximides.
