# Supplementary material for: New Thalidomide-Resembling Dicarboximides Target ABC50 Protein and Show Antileukemic and Immunomodulatory Activities
Source: Biomolecules. 2019 Sep 4;9(9):446. doi: 10.3390/biom9090446 (PMC6770581; doi:10.3390/biom9090446)
Supplement: Supplementary file 1 [file biomolecules-09-00446-s001.zip › Cieslak Supplementary TabS1 FigS1 FigS2.docx]

**Supplementary Material**

**Table S1**. Tests and cell lines used to characterize the biological activity of dicarboximides. The numbers correspond to compounds listed in Table 1.

|  | Cytotoxicity | Apoptosis | Expression of proapoptotic genes | Pull-down assay | IKZF1/IKZF3 assay | ABC50 PROTAC |
| --- | --- | --- | --- | --- | --- | --- |
| HeLa | 1-9 |  |  |  |  | 7-9 |
| K562 | 1-9 | 3, 5, 6 | 3, 6 | 6b | 3, 5, 6 | 7-9 |
| HL-60 | 1-6 |  |  |  |  |  |
| HUVEC | 1-9 |  |  |  |  |  |
| MOLT-4 | 1-9 | 1-6 |  |  | 3, 5, 6 |  |
| CFPAC | 1-6 |  |  |  |  |  |

**Figure S1.** The numbering of atoms of derivative 6b used in the description of nuclear magnetic spectra.

**Figure S2.** The numbering of atoms of compounds 7-9 used in the description of nuclear magnetic spectra。
